# Metacognition for Listening in Noise: The Role of Age and Hearing Loss

**DOI:** 10.1097/AUD.0000000000001798

**Published:** 2026-03-09

**Authors:** Chiara Valzolgher, Elena Giovanelli, Elena Gessa, Tommaso Rosi, Giulia Pasin, Rebecca Ferrari, Matteo Pegoraro, Francesco Pontoni, Domenico Spinella, Giuseppina Tromballi, Giuseppe Nicolò Frau, Francesco Pavani

**Affiliations:** 1 Center for Mind/Brain Sciences – CIMeC, University of Trento, Rovereto, Italy; 2 LEVEL up, Laboratorio di Comunicazione Scientifica e Progettazione Didattica, Trento, Italy; 3 Department of Psychology and Cognitive Sciences (DiPSCo), University of Trento, Italy; 4 Pontoni udito e Tecnologia, Trento, Italy; 5 Studio UdiVer, Mori, Italy; 6 Unità Operativa di Otorinolaringoiatria Ospedale Santa Maria del Carmine Rovereto APSS Trento, Rovereto, Italy; 7 Centro Interuniversitario di Ricerca “Cognizione, Linguaggio e Sordità” – CIRCLeS, Trento, Italy; 8 These authors contributed equally to this study.

**Keywords:** Age-related hearing loss, Hearing in noise, Locus of control, Metacognition, Self-efficacy

## Abstract

**Objectives:**

Despite general awareness about hearing difficulties in aging, older adults often respond to listening challenges by adopting maladaptive coping strategies, such as social withdrawal or isolation, that could negatively affect their well-being. Reasons behind such behaviors have been studied within the framework of metacognition (i.e., the ability to monitor one’s performance and reflect on one’s cognitive processes), which plays an important role in shaping strategic behavior. While previous research suggests that metacognitive abilities for listening are preserved with age, studies with younger adults indicate that hearing loss (HL) might reduce self-efficacy and perceived control over listening challenges, potentially influencing strategy use. Whether similar effects occur in older adults, a population particularly affected by listening difficulties, remains unclear. This study examined the impact of age and mild HL on metacognitive abilities related to listening and studied their association with the use of compensatory strategies.

**Design:**

Seventy-nine participants were recruited: 26 younger adults with normal hearing (NH; 20.8 yrs; 3 males), 26 older NH adults (64.6 yrs; 8 males), and 27 older adults with HL, none of whom used hearing aids (67.7 yrs; 14 males). Metacognitive monitoring was assessed through a listening-in-noise task, and broader metacognitive constructs (self-efficacy, locus of control, and knowledge of listening abilities) were evaluated using questionnaires. In addition, the influence of age and HL on self-reported use of compensatory strategies (tested by using questionnaire) was examined, along with their relationship with metacognitive factors. Group comparisons were performed using parametric *t* test analyses.

**Results:**

Age alone did not affect metacognitive monitoring, nor beliefs and knowledge about listening abilities. However, older adults with HL reported lower self-efficacy and less frequent use of some behavioral strategies compared with their NH peers, in the context of intact monitoring skills. Notably, perceived control and self-efficacy were associated with reported strategy use, especially among participants with HL.

**Conclusions:**

Even mild HL appears to be reflected in older adults’ self-perceptions of listening abilities. The links between metacognitive beliefs, perceived control, and strategy use suggest that these factors may shape coping behavior in the presence of HL. Our findings highlight the importance of addressing metacognitive aspects in interventions aimed at promoting adaptive listening strategies among older adults with hearing loss.

## INTRODUCTION

In daily life, we frequently find ourselves in noisy environments, such as crowded restaurants or bustling streets. In these contexts, what reaches our ears is a complex mixture of sound streams that our cognitive system must separate and process to make sense of the auditory scene (Bregman 1994; Sussman 2017). This process relies on the quality of auditory input (bottom-up processing), but also on the efficiency of cognitive functions, such as attention and working memory (top-down processing; Bronkhorst 2015). This interplay has been described, for instance, in the Ease of Language Understanding (ELU) model (Rönnberg et al. 2013, 2019, 2022): when the auditory input is degraded, listeners rely more heavily on explicit cognitive resources to compensate for mismatches between input and stored representations. Among these explicit resources, top-down cognitive processes, such as attention, working memory, and the use of prior knowledge, may support or interfere with successful listening.

The consequences of age-related declines in both auditory and cognitive functions for understanding speech in noise have been examined extensively (Salvi et al. 1983; Pichora-Fuller 1997; Glyde et al. 2011; Rosemann & Thiel 2020; Slade et al. 2020; Fostick & Schneider 2022; Rönnberg, 2003). However, to what extent aging adults are aware of their listening skills and control their behavior in response to this listening awareness remained largely underinvestigated. The general psychological construct that is relevant in this context is metacognition, generally defined as the ability to think about one’s own cognition (Flavell 1979). It encompasses the monitoring of performance outcomes (metacognitive monitoring), knowledge about cognitive processes and strategies (metacognitive knowledge), and beliefs regarding one’s abilities in specific cognitive domains, such as self-efficacy (SE) and perceived control over cognitive challenges. These components jointly contribute to shaping how individuals regulate and adapt their behavior in response to cognitive demands (Efklides 2008).

Within the influential framework proposed by Nelson and Narens (1990), metacognition involves a dynamic interaction between two levels: the level where cognitive operations occur (termed object-level), and the level which monitors and regulates these cognitive operations as they unfold (termed meta-level). Monitoring processes allow individuals to evaluate ongoing performance (e.g., confidence, perceived effort) (Pichora-Fuller et al. 2016), whereas control processes guide subsequent decisions or behavioral adjustments. Building on this framework, Koriat and colleagues (Koriat & Goldsmith 1996; Koriat et al. 2006) emphasized that the accuracy of monitoring directly influences the effectiveness of control. In the context of listening, accurate monitoring of one’s comprehension and listening confidence should therefore support the adaptive regulation of behavior such as the decision to use compensatory strategies or seek environmental support (Dunlosky et al. 2003).

A clearer understanding of metacognition in listening among older adults may help close the gap between their awareness of listening challenges in noise (D’Haese et al. 2018) and the maladaptive strategies they often use, such as communication withdrawal and social isolation (Shukla et al. 2020), which can adversely affect quality of life and elevate risks of cognitive decline (Livingston et al. 2017, 2020) and depression (Lawrence et al. 2020).

### The Role of Age on Metacognition for Listening

Research on the effects of aging on metacognition has mainly focused on metamemory, examining metacognitive monitoring, knowledge, and self-beliefs. However, findings in this domain are not univocal. For instance, some studies suggest that metacognitive monitoring for memory declines with age (Touron et al. 2010), while others report that it remains stable in older adults (Rawson et al. 2011). Similarly, evidence on memory SE (i.e., the perceived ability to perform in a cognitive domain; Bandura 1977; Tarricone 2011) is mixed. Some research indicates a decline with age in memory SE (Marques et al. 2020) and monitoring (Pearman & Storandt 2004; Pansky et al. 2009), whereas other studies have found higher levels of memory SE in older adults compared with younger individuals (Luszczynska et al. 2005). Contradictory findings also emerge regarding locus of control (LoC), defined as the degree to which individuals perceive their memory performance as dependent on internal versus external factors (Scarampi & Gilbert 2021). While some studies report that older adults tend to attribute outcomes to external factors like age or luck (Hess et al. 2003), others suggest they maintain an internal LoC by attributing outcomes to controllable causes like effort or motivation (Lachman 1986).

Metacognition in the context of listening in noise, hereinafter defined as metalistening, has only recently been investigated. Some studies suggest that metalistening can be preserved in older adults (Giovanelli et al. 2024, 2025; Valzolgher et al. 2024). However, other studies investigating metacognitive monitoring for listening showed less efficiency for older adults, especially when top-down listening processes are involved. For instance, Rogers et al. (2012) studied the phenomenon of false hearing. This phenomenon refers to high-confidence listening errors made upon semantic predictability of a to-be-heard word, and is believed to occur when semantic predictability is preferred over a more effortful phonological analysis of the auditory input (e.g., hearing the word HAY associated with the word BARN, while the actual to-be-heard word was PAY). Older adults tend to make more false hearing errors, suggesting that performance monitoring might become less effective with age. However, it cannot be excluded that false hearing is instead fostered by older adults’ ineffective choices of efficient listening strategies (i.e., choosing a top-down semantic strategy over a bottom-up, more stimulus-driven strategy).

A further weakness in metalistening abilities of older adults appears to emerge when they are asked to describe how they perceive the listening experience of themselves or other people. Valzolgher et al. (2025) asked participants to assess the effort required in different listening situations for themselves and for three different characters: a young adult, an older adult, and an older adult with hearing loss (HL). The results showed that older adults perceive their listening effort as smaller from that of their ingroup (the older adult character), whereas a group of young adults judges their listening effort as very similar to that of their ingroup (the young adult character). The authors interpreted this finding as initial evidence that older adults may have a distorted representation of their own listening effort.

In sum, the impact of aging on metalistening remains largely underinvestigated, with current evidence showing mixed results that appear to vary depending on experimental demands. This underscores the importance of replicating and extending these observations.

### The Role of HL on Metalistening in Older Adults

When addressing the impact of aging on metalistening, it is important to consider that around 30% of individuals over 60 yrs of age experience HL (World Health Organization 2025), making it the most common form of sensory decline among older adults (Roth et al. 2011). This sensory degradation in sensory input places an additional demand on cognitive functions that support comprehension, such as attention and working memory (Rönnberg et al. 2013, 2019, 2022; Bronkhorst 2015).

In such a context, knowing and monitoring listening difficulties, as well as implementing effective listening strategies, becomes extremely relevant for older individuals experiencing HL. Evidence of the influence of HL on metacognitive components comes from studies targeting self-beliefs in young HL individuals. For example, Cuevas et al. (2019) found that general LoC predicts the perception of general SE in HL individuals. Crucially, individuals with HL had lower SE as compared with normal hearing (NH) peers highlighting the impact HL can have on self-assessment. Dammeyer et al. (2018) examined personality traits and SE among deaf and hard-of-hearing college students, both with and without cochlear implants. Hard-of-hearing individuals exhibited lower SE compared with their NH peers, again pointing to a role of hearing deficits in shaping internal beliefs and, ultimately, behavior. These findings suggest that HL may negatively influence self-beliefs and coping strategies, particularly in older adults who already face challenges related to sensory decline and increased cognitive demands. In this context, HL could undermine metacognitive functioning, such as the ability to monitor performance and maintain confidence, ultimately affecting behavioral responses to listening difficulties. Moreover, given that age-related HL is often accompanied by negative social stereotypes (e.g., associations with cognitive decline or incompetence; Barber & Lee 2015), older adults with HL may be especially vulnerable to reduced SE and perceived control (Oyler 2012). In this perspective, the negative coping may be influenced by the presence of an HL, rather than upon age alone.

### Study Aim

Building on the literature reviewed earlier, this study aims to systematically investigate how aging and HL affect metacognitive abilities related to listening in noise, and how these factors relate to the use of compensatory strategies. Specifically, we address three research questions.

The first aim is to examine whether and how age influences metalistening abilities in young and older adults. Previous evidence (Giovanelli et al. 2024) suggests that different facets of metacognition, including monitoring abilities, knowledge of listening difficulties, and self-beliefs, may be preserved with age, at least in individuals with NH. Here, we aim to replicate and extend these findings to clarify whether age has a negligible, negative, or even positive effect on metalistening abilities.

The second aim is to determine whether age-related HL affects metacognition for listening in noise. To this end, we compared older adults with and without HL. We predicted that HL would negatively impact metalistening, particularly perceived control over listening challenges (LoC) and listening SE. Such difficulties could reduce the accuracy of self-assessment during the hearing-in-noise task, leading to less effective metacognitive monitoring.

The third and more exploratory aim is to assess whether age and HL are associated with the use of compensatory strategies, and whether these behaviors are linked to individual differences in metacognitive abilities. By compensatory strategies, we refer both to adaptive and maladaptive behavioral responses to difficult listening situations (Demorest & Erdman 1986) and to attitudes toward hearing aid adoption (Adorni et al. 2022). Adaptive behaviors include orienting toward the sound source, using visual cues, or reducing background noise. We expected a decrease in adaptive behaviors and an increase in maladaptive behaviors with aging, as suggested by studies on metamemory (Borkowski et al. 1987; Souchay & Isingrini 2004; Hertzog & Dunlosky 2011).

As part of this third aim, we also considered hearing aid uptake as an additional compensatory strategy, we hypothesize that better metalistening abilities will be associated with a greater use of adaptive strategies and a more favorable decision to adopt hearing aids. Previous studies have identified several factors influencing hearing aid uptake, such as familial (e.g., family support), social (e.g., access to services), economic (e.g., costs), and sensory aspects (e.g., degree of HL) (Gomez & Madey 2001; Alvarado 2011; for reviews: Knudsen et al. 2010; Knoetze et al. 2023). However, no study has examined whether metacognitive abilities also play a role in this decision. We propose that hearing aid adoption can be conceptualized as an adaptive strategy, with higher metacognitive awareness predicting a greater likelihood of adoption. Alternatively, individuals with strong metacognitive skills might consider hearing aids as a last-resort solution, using them only when other compensatory strategies prove insufficient.

## MATERIALS AND METHODS

### Participants

Seventy-nine participants were recruited for the study, divided into 3 groups: 26 younger adults with NH, 26 older adults with NH, and 27 older adults with mild HL. None of the participants with HL used hearing aids. The sample size was determined based on previous studies investigating metalistening in older adults, which included comparable group sizes (Scherer & Frisina 1998; Ben-David et al. 2012; Besser et al. 2015; Giovanelli et al. 2024, 2025 [30 per group]; [20 per group]; [20 per group]; [30 per group]; [26 per group]). The sample of older adults included individuals from a broad geographic area, reducing the risk of sampling bias associated with a single metropolitan area (see also Hearing in Noise Task for details). We intentionally recruited older adults with only mild HL to avoid including individuals already using hearing aids and to focus on those at the earliest stages of hearing decline.

The younger adult group had a mean age of 20.8 yrs (SD = 1.9, range = 19 to 25; 3 males). The older adult groups had mean ages of 64.6 yrs (SD = 3.4, range = 60 to 76; 8 males) for the NH participants, and 67.7 yrs (SD = 5.2, range = 56 to 77; 14 males) for the HL participants. Although most participants in the HL group were aged 60 or older, 2 individuals were slightly younger (aged 56 and 57). Because these ages are commonly considered representative of the older adult population in previous studies (Monteiro de Castro Silva & Feitosa 2006; Helfer & Freyman 2014), they were retained in the group. All participants were Caucasian, native Italian speakers, with normal or corrected-to-normal vision, and no cognitive deficits as assessed by the Montreal Cognitive Assessment, applying the age- and education-adjusted correction recommended by Santangelo et al. (2015).

Audiometric thresholds were assessed separately for each ear using a standard ascending-descending pure-tone audiometry (PTA) procedure, either assessed by audiometrists at collaborating audiology centers (Hospital of Santa Maria del Carmine, Rovereto; Pontoni Udito e Tecnologia, Trento; Studio UdiVer, Mori) or by the experimenter on the day of testing. Hearing thresholds were classified according to the World Health Organization (2021) criteria and Italian national guidelines for hearing aid provision. Pure-tone audiometry were calculated across 500, 1000, 2000, and 4000 Hz. Thresholds up to 20 dB HL were considered NH, while those up to 35 dB HL were classified as mild HL. Group-averaged audiometric thresholds are reported in Supplementary Figure 1, Supplemental Digital Content, https://links.lww.com/EANDH/B845. Hearing thresholds were measured using calibrated audiometers. The experimenter used an Inventis Harp audiometer (frequency range: 125 to 8000 Hz; EN 60645-1/ANSI S3.6, Type 2; intensity range: air conduction −10 to 120 dB HL, bone conduction −10 to 80 dB HL). Pontoni Udito e Tecnologia also used an Inventis Harp audiometer (same technical specifications), while Studio UdiVer used an Amplaid AM11 audiometer (EN 60601-1; TDH-49 transducers; air conduction −10 to 120 dB HL).

### Stimuli

#### Hearing in Noise Task

Speech stimuli for the experiment comprised 20 words selected from the Italian version of the Matrix Sentence Test used for assessing hearing-in-noise abilities (Puglisi et al. 2015). Following the approach of ), four-word phrases were created to reduce memory load. The stimuli were identical to the ones used by Giovanelli and categorized into four lists involving names, verbs, numerals, and objects (e.g., “Luca compra quattro pietre,” which translates in “Luca buys four rocks”). A young male speaker pronounced the words by emulating the characteristics of the original Matrix test. Specifically, the speaker maintained a consistent voice level and a 1-sec pause between words. Recordings were conducted in a soundproof booth (Amplifon G2 × 2.5; floor area 200 × 250 cm, height 220 cm) using a video camera (Sony HDR-PJ420) and a microphone (Sennheiser E835). The audio and visual tracks were recorded simultaneously, enabling better segmentation for articulation. Post-recording, tracks were cleaned to eliminate errors and occasional noises (DaVinci Resolve 17). The final audio tracks, with uniform voice levels and a high-pass filter, were used to create 60 unique 4-word phrases for the experiment. The background noise (63 dB), resembling crowded environments, was generated by multiple voices without music (Audacity 2.4.1). Given that the stimuli used were the same as those used in the experiment of Giovanelli et al. (2024), refer to that article for detailed information.

All stimuli were presented within a VR environment controlled by a Unity-based script. Verbal responses were recorded by the experimenter via mouse input. Each trial consisted of a four-word sentence spoken by a male voice, presented against a background of informational noise. For every trial, a segment of noise was extracted from the beginning of the noise track and matched in duration to the target sentence, with an additional 500 msec appended before and after the sentence. Because the duration and content of the sentences varied, the overlap between target and noise differed across trials. However, a fixed interval between the noise onset and the sentence onset was maintained to help participants detect the target phrase within the noise.

#### Questionnaires

##### Questionnaire about metacognitive aspects

###### Listening Challenges Attitude Scale

Listening Challenges Attitude Scale (LiCAS; Giovanelli et al., Reference Note 2) was adopted to assess two main components of metalistening experience: LoC and SE. The LoC component of LiCAS includes two complementary subscales: the hearing loss likelihood (HLL) and the control over hearing loss (CHL) (Giovanelli et al., Reference Note 2).

The LoC-HLL subscale measures individuals’ beliefs about the likelihood of having or developing HL. It includes six items, each of which presents a statement that participants rate based on their level of agreement. An example item is, “I think there’s a good chance I will develop hearing loss in the future.” Higher scores on this subscale indicate a more external perception of control over listening outcomes. Because the LoC-HLL subscale was originally designed to capture LoC in hearing individuals some items explicitly refer to hearing problems (e.g., “Sometimes I think I might have a hearing problem”; “When I don’t understand something, I tend to think I have a hearing problem”). However, other items address LoC aspects related to communication, without explicitly mentioning hearing problems (e.g., “No matter how much I want it, I can’t understand well”; “If people around me don’t help, I struggle to understand what is said in conversation”). The LoC-CHL subscale assesses participants’ perceived ability to improve hearing issues. Comprising three items, it measures how much control participants believe they have over their hearing experience. Higher scores here indicate a more internal perception of control over listening.

The SE component of LiCAS consists of two additional subscales: self-efficacy in easy listening conditions (SE-Easy) and the complex listening conditions (SE-Hard). The SE-Easy subscale (five items) evaluates confidence in understanding speech in relatively straightforward listening situations. The SE-Hard subscale (four items) assesses SE in more challenging listening environments. Each item in the SE subscales describes a specific listening scenario where comprehension is required, and participants are asked to rate their confidence in understanding conversation in that situation. For instance, one item reads: “I can understand one-on-one conversation in a quiet place when unable to see the speaker’s face” (see entire list of items in Supplementary Table 2, Supplemental Digital Content, https://links.lww.com/EANDH/B845). Responses were rated on a 10-point scale (1 = strongly disagree, 10 = strongly agree), expressing the degree of agreement with each statement.

###### Metacognitive knowledge questionnaire

Developed by Giovanelli et al. (2024), this questionnaire was designed to assess metacognitive knowledge about listening difficulties across a range of situations. It was adapted from the Speech subscale of the Speech, Spatial, and Qualities of Hearing Scale (SSQ) originally created by Gatehouse and Noble (2004). The items are phrased in the third person to generate a measure of general knowledge that is less influenced by respondents’ personal abilities, focusing instead on broader understanding and perceptions of listening difficulties rather than individual SE. The questionnaire consists of 14 items organized into 2 subscales: easy listening scenarios (7 items) and difficult listening scenarios (7 items). Scores are calculated as the average of relevant items for both the total and subscale scores, with responses measured on a scale from 0 to 100.

###### Hearing aids questionnaire

As in the study of Adorni et al. (2022), the attitude toward hearing aids was assessed by using a semantic differential item (Maggino & Mola 2007) on a seven-point scale. Participants were asked to express if hearing aids could be described by using the adjective not useful (1) or useful (7) or choosing a possibility in between (“Which adjective do you think is the most suitable to describe hearing aids?”). Higher scores reflected a positive attitude. The behavioral intention to adopt hearing aids was also measured. As in the study of Adorni et al., participants were asked to indicate their agreement on a five-point scale, ranging from 1 (not at willing) to 5 (very willing) in response to the sentence “If you experience deterioration or hearing loss now or in the future, indicate how likely you are to purchase hearing aid.”

##### Questionnaire investigating strategic aspects

###### Coping strategies for listening challenges

For the assessment of coping strategies in response to listening challenges, participants were asked to report the frequency of their use of various behavioral strategies on a five-point scale ranging from 1 (rarely) to 5 (almost always). All items from the Communicative Profile for the Hearing Impaired (CPHI, Demorest & Erdman 1987) were adapted and translated into Italian. This 20-item questionnaire is divided into 3 subscales: (1) Maladaptive Strategies subscale (6 items) reflects maladaptive responses to everyday listening situations in noise, describing unhelpful reactions people might adopt. For instance, one item states, “When someone I asked to repeat something gets angry, I stop asking them to repeat.” (2) Verbal Strategies subscale (6 items) focuses on adaptive verbal techniques for managing challenging listening situations, such as clarifying missed information. An example item is, “If I do not understand the first repetition, I ask the person to repeat what they said.” (3) Non-Verbal Strategies subscale (8 items) explores nonverbal approaches that could improve listening effectiveness. One example is, “When I have difficulties hearing, I focus on the face of the person who is talking to me” (see Supplementary Table 3, Supplemental Digital Content, reporting all items, https://links.lww.com/EANDH/B845).

###### Coping strategies for HL

In this questionnaire, participants rated on a scale from 1 (rarely) to 5 (almost always) how frequently they use certain strategies. The questions varied based on whether participants reported having HL. Those with hearing difficulties responded to five items about how their condition affects social interactions (e.g., “I avoid talking to strangers because of hearing loss”). Participants without HL answered five hypothetical questions about potential future difficulties (e.g., “If I have a hearing problem in the future, I will avoid social situations”) (see Supplementary Table 4, Supplemental Digital Content, https://links.lww.com/EANDH/B845).

### Apparatus

For the experimental task, a Meta Quest 2 (256GB; resolution: 3616 × 1840; frequency: 72 Hz) linked to a computer (ASUS TUF Dash F15) via an Oculus link cable to display the VR environment was used. This technology was combined with a headphone (Sennheiser HD 650 S; HiFi, frequency range: 10 to 41.000 Hz) to deliver the sounds. Sounds were not spatialized, nor mimicked room reverberation.

### Procedure

**Fig. 1.**
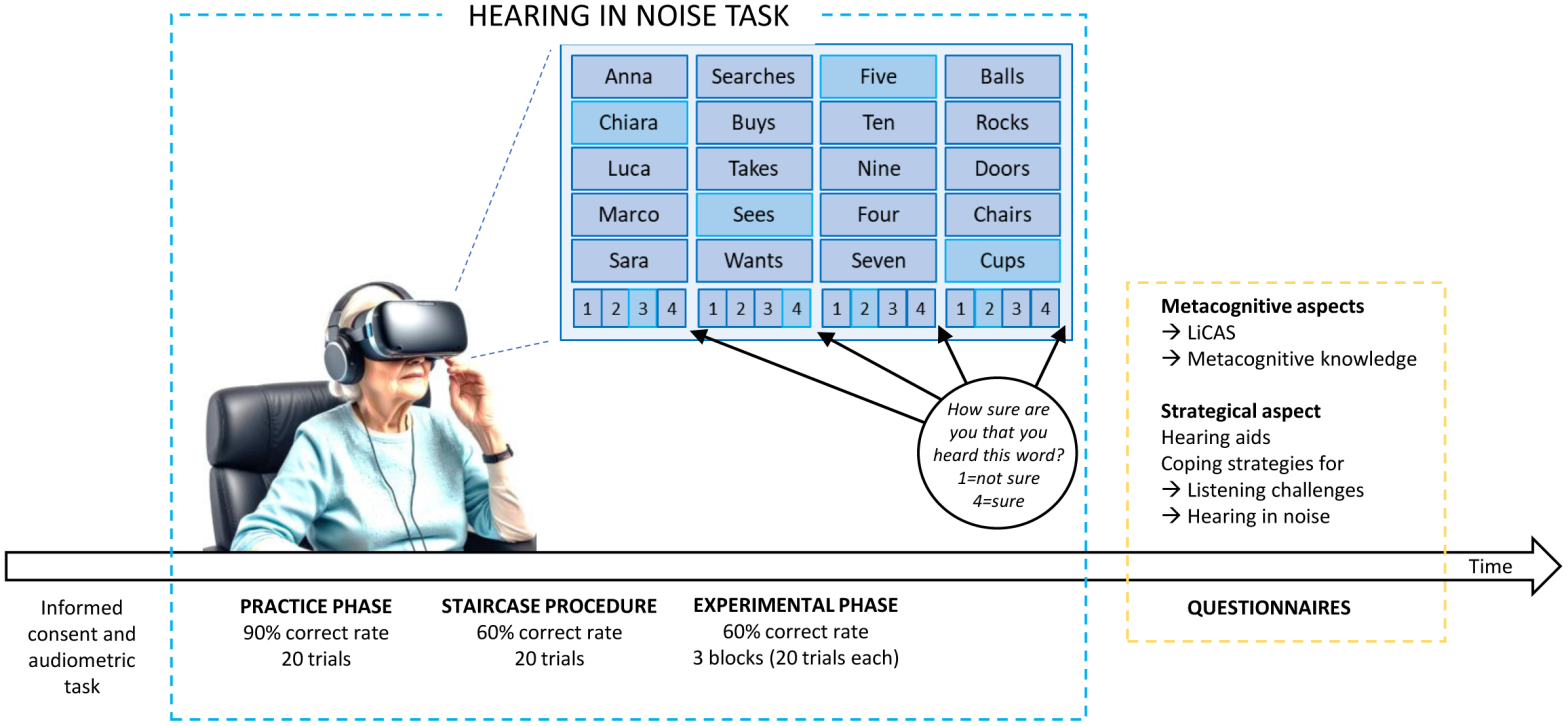
Experimental timeline and procedure. Overview of the study sequence: participants provided informed consent, completed speech, and pure-tone audiometry, and performed a hearing-in-noise task consisting of practice, staircase, and task including three experimental blocks under two listening conditions. Finally, they completed questionnaires on metacognitive and strategic aspects. The blue rectangle shows the answer matrix presented to participants after each phrase; original Italian labels are translated for clarity. LiCAS indicates Listening Challenges Attitude Scale.

As illustrated in Figure 1, participants began the experimental session after providing informed consent. Next, they completed the cognitive assessment (Montreal Cognitive Assessment, Santangelo et al. 2015) and, if not previously conducted, the PTA test. Finally, participants performed the hearing in noise task and, at the end of the session, completed a series of questionnaires (see Questionnaires for details).

#### Hearing in Noise Task

Before starting the experimental task, participants received instructions regarding the VR environment and the task they would perform. The experimenter assisted participants in putting on the VR headset and headphones, ensuring proper fit and clear visual perception of the virtual scene to avoid any blurring. The virtual environment depicted a minimalistic white rectangular room (3 × 4 m) devoid of distinguishing features. To mitigate any potential sense of confinement, a virtual door was positioned behind the participant. A welcome screen appeared in front of them at the start of the session (Fig. 1). During the task phases, participants verbally reported the words they heard for each trial, selecting them from a matrix displayed on the virtual screen in front of them. Notably, the matrix was only visible at the end of each trial when participants were required to provide their responses and was not displayed during the listening phase (Fig. 1). For a better understanding of the participant’s view within the VR apparatus, see the video available at https://osf.io/atcv9/.

The VR experiment began with a practice phase, during which participants were asked to identify spoken words with an expected accuracy of approximately 0.9 proportion correct. This phase, consisting of 20 four-word phrases presented in noise, aimed to familiarize participants with the task dynamics; data from this phase were not included in the analyses. The second phase used an adaptive staircase procedure based on the method described by Brand and Kollmeier (2002), calibrated for four-word phrases with a target performance of 0.6 proportion correct word recognition (as in Giovanelli et al. 2024). The signal to noise ratio (SNR) was adjusted by modifying only the target voice level, starting from +4.9 dB. This procedure allowed for the individual calibration of task difficulty, ensuring comparable performance across participants and reducing ceiling or floor effects that could compromise the assessment of metacognitive monitoring. In the experimental phase, participants completed 3 experimental blocks of 20 phrases each (a total of 60 phrases), presented at the SNR established during the staircase phase. After each phrase, they selected the words they believed they had heard from a response matrix. In addition, participants rated their confidence in each selected word on a four-point scale (1 = “Not at all sure” to 4 = “Completely sure”). If uncertain, they were instructed to make a forced-choice selection. Breaks were permitted between blocks to minimize fatigue. All participants completed the VR task successfully, with the exception of one (ID = 42), who partially performed the task without the headset due to discomfort. Throughout the experiment, participants’ verbal responses were recorded by the experimenter using a computer mouse.

After completing the hearing-in-noise task, participants filled out a set of online questionnaires outside the VR environment, using a computer and mouse. They first provided demographic information and details about their hearing status (e.g., hearing aid experience, presence of tinnitus), followed by questionnaires assessing metacognitive aspects. These included (1) the LiCAS, covering LoC and SE; (2) the Metacognitive Knowledge Questionnaire; and (3) the Hearing Aid Questionnaire. Finally, participants completed two questionnaires on compensatory strategies: (1) Coping Strategies for Listening Challenges and (2) Coping Strategies for HL (see Questionnaire About Metacognitive Aspects).

### Analyses

All analyses were conducted using JASP (version 0.17.1.0) and R (version 4.4.1). We adopted a general analytical framework designed to ensure comparability across measures while limiting redundancy. Parametric tests (independent-sample *t* tests, repeated-measures analysis of variance [ANOVA]) were used when assumptions were met. When the assumption of homogeneity of variance was violated, Welch’s correction was applied. For repeated-measures ANOVA, Greenhouse–Geisser correction was used where sphericity was violated. To assess the strength of evidence for or against the null hypothesis, Bayesian analyses were also conducted. Bayes factors (BF_10_) are reported alongside *p* values, with values above 3 indicating moderate evidence and values above 10 indicating strong evidence for the alternative hypothesis. Where multiple dependent variables were tested within a conceptual family (e.g., questionnaire subscales), we applied Holm–Bonferroni corrections to control for inflated type I error. Relationships between continuous variables were examined using Spearman’s rank correlations. Regression analyses were conducted to test directional hypotheses based on prior literature (Cuevas et al. 2019).

Metacognitive monitoring was defined as the correspondence between participants’ confidence ratings and their actual accuracy, reflecting the ability to discriminate correct from incorrect responses on a trial-by-trial basis (Nelson & Narens 1990; Giovanelli et al. 2024). Among the several indices used to capture this correspondence, we selected the area under the type 2 receiver operating characteristic curve (AUC), which provides a bias-corrected estimate of metacognitive sensitivity (Fleming & Lau 2014). Compared with nonparametric correlations such as gamma (Masson & Rotello 2009), the AUC metric is less affected by response tendencies or restricted variability in confidence ratings, and is therefore particularly appropriate for comparing groups that may differ in confidence use. This trial-level measure of monitoring is conceptually distinct from the global metacognitive judgments captured through our questionnaires, which reflect participants’ general self-beliefs about listening ability and control (Schraw 1994).

When conducting analyses on the effect of age on metacognition (see The Role of Age on Metalistening), data from Giovanelli et al. (2024) were also included, taking advantage of the full methodological overlap between studies. This merged analysis was conducted exploratorily to verify whether previously null effects could reflect sample size limitations, rather than being theoretically driven.

All supplementary analyses are explicitly referenced in the Results section and reported in detail in the Supplementary Materials, Supplemental Digital Content, https://links.lww.com/EANDH/B845.

## RESULTS

All analyses followed the general framework described in Analyses. Parametric or Welch-corrected *t* tests and ANOVAs were used depending on the assumption of equal variances, with Holm–Bonferroni correction applied to control for multiple comparisons. Bayesian analyses (JASP v0.17.1.0) were conducted in parallel to quantify evidence for or against the null hypothesis, and Bayes factors (BF_10_) are reported where relevant.

The results are presented in two main sections. First, the effect of age was analyzed by comparing younger and older adults with NH (see The Role of Age on Metalistening). Then, the effect of hearing ability was examined by comparing older adults with NH to those with mild HL (see The Role of Hearing Loss in Older Adults on Metalistening).

### The Role of Age on Metalistening

Young and older participants with NH (26 in each group) differed in age (NH young: M = 20.8, SD = 1.9, range = 19 to 25; NH older: M = 64.6, SD = 3.4, range = 60 to 76; *t*(50) = 57.4, *p* < 0.001, BF_10_ = 2.29 × 10^43^). Both groups met the criteria for NH (see Supplementary Figure 1, Supplemental Digital Content, https://links.lww.com/EANDH/B845), although with a significant overall difference in PTA (averaged across 500, 1000, 2000, and 4000 Hz): NH young = 5.6 dB HL (SD = 4.2, range = −0.6 to 16.3), NH older = 15.0 dB HL (SD = 3.9, range = 8.1 to 20.6; *t*(50) = 8.31, *p* < 0.001, BF_10_ = 1.14 × 10^8^).

#### Hearing in Noise Task: Performance, Confidence, and Metacognitive Monitoring

**Fig. 2.**
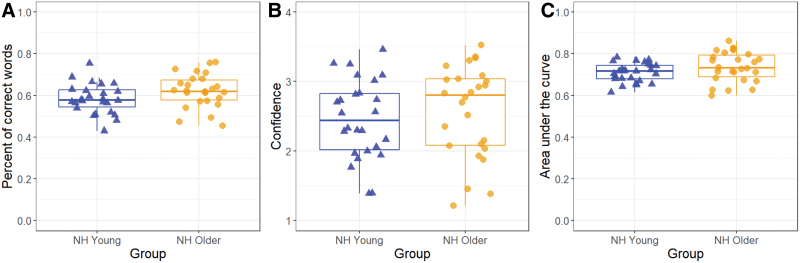
Hearing in noise task and metacognitive monitoring—NH young and NH older. Upper panel: hearing-in-noise task. (A) Percent of correct words, (B) mean confidence, and (C) metacognitive monitoring (area under the curve) values for the two groups: young (triangles) and older adults with NH (circles). Values are reported in Supplementary Table 5, Supplemental Digital Content, https://links.lww.com/EANDH/B845. NH indicates normal hearing.

Following the staircase procedure aiming for approximately 0.6 proportion correct, performance in the hearing in noise task was comparable in the two groups (NH young: 0.62 ± 0.08; NH older: 0.59 ± 0.07, *t*(50) = 1.73, *p* = 0.09, *d* = 0.48, BF01 = 1.07; Fig. 2A). Groups were also comparable in terms of confidence (NH young: 2.52 ± 0.63; NH older: 2.71 ± 0.50, *t*(50) = 1.26, *p* = 0.21, *d* = 0.35, BF_10_ = 1.87; Fig. 2B) and when considering the AUC values as index of metacognitive monitoring (NH young: 0.74 ± 0.07; NH older: 0.71 ± 0.05, *t*(Welch) (41.96) = 1.63, *p* = 0.11, *d* = 0.45 BF01 = 1.21; Fig. 2C).

Consistent with the analyses proposed by Giovanelli et al. (2024), the correlation between percent corrects and confidence was examined. In both groups, participants with better accuracy were also those who reported higher confidence (NH young: *R* = 0.54, *p* = 0.005; NH older: *R* = 0.61, *p* < 0.001). For completeness, we also examined the effect of word order as in Giovanelli et al. (see Supplementary Results, Supplemental Digital Content, https://links.lww.com/EANDH/B845).

To increase the robustness of these findings and assess whether the absence of group effects might reflect limited statistical power, we exploratorily integrated data from Giovanelli et al. (2024), which used the same design and procedure (note that considering the AUC analysis reported by Giovanelli et al., the integration of datasets increased the statistical power from 0.41 to 0.63. The combined dataset included 105 participants (56 young: M = 22.8 ± 3.1; 49 older: M = 65.8 ± 2.8). Three separate *t* tests were then conducted on performance, confidence, and monitoring accuracy. The analyses on performance and confidence confirmed the absence of significant differences between young and older adults (*t*(103) = 0.42, n.s., *d* = 0.08; *t*(103) = 1.11, n.s., *d* = 0.22), whereas the analysis on metacognitive monitoring revealed higher accuracy in older adults compared with younger participants (*t*(103) = 2.27, *p* = 0.025, *d* = 0.45).

#### LoC, SE, and Metacognitive Knowledge

The effect of age on subscales of LiCAS and metacognitive knowledge (easy and hard) was examined using parametric tests and Bayesian statistics. Means with SD and statistical outcomes are reported in Table 1. None of these comparisons reached statistical significance (all uncorrected *ps* > 0.35).

**TABLE 1.** Questionnaires about metacognitive aspects—NH young and NH older

	Groups Means		Statistics	
Indices	NH Young	NH Older	*t* Test	BF
LiCAS-LoC				
HLL (LoC-HLL)	2.89 ± 1.48	2.99 ± 1.77	t(50) = 0.22, n.s., *d* = 0.06	BF*01 = 3.52*
CHL (LoC-CHL)	5.85 ± 2.11	5.60 ± 2.65	t(50) = 0.37, n.s., *d* = 0.10	BF*01 = 3.40*
LiCAS-SE				
Easy (SE-easy)	9.34 ± 0.61	9.52 ± 0.73	t(50) = 0.95, n.s., *d* = 0.26	BF*01 = 2.49*
Hard (SE-hard)	8.25 ± 1.15	8.20 ± 1.21	t(50) = 0.15, n.s., *d* = 0.04	BF*01 = 3.56*
Mean	8.77 ± 0.70	8.76 ± 0.85	t(50) = 0.06, n.s., *d* = 0.02	BF*01 = 3.59*
Metacognitive knowledge				
Easy	2.62 ± 1.23	2.76 ± 1.38	t(50) = 0.41, n.s., *d* = 0.11	BF*01 = 3.35*
Hard	5.56 ± 1.23	5.31 ± 1.84	t(50) = 0.57, n.s., *d* = 0.16	BF*01 = 3.14*

Mean, SD, and statistics (*t* tests) of LiCAS (SE, LoC) and metacognitive knowledge.

BF, Bayes factors; CHL, control over hearing loss; HLL, hearing loss likelihood; LiCAS, Listening Challenges Attitude Scale; LoC, locus of control; NH, normal hearing; SE, self-efficacy.

These data also offered the opportunity to explore the relationships between SE and LoC, hypothesizing a directional link between these two variables (Cuevas et al. 2019). To this aim, a regression analysis with LoC-HLL as predictor and the SE mean index as the dependent variable, including group (young adults and older adults with NH) as an interacting factor was conducted. The results revealed that a more external LoC (i.e., higher LoC-HLL values) was associated with lower SE (estimate = −0.24, *t* = −3.17, *p* = 0.003). Yet, no main effect or interaction involving group was observed (all *p*s > 0.79). No effects were found when testing LoC-CHL as a predictor (all *p*s > 0.88).

#### Behavioral Strategies

The effect of age on behavioral strategies (subscales of Coping Strategies for Listening Challenges, Coping Strategies for Hearing Loss, Attitude Toward Hearing Aids, and Behavioral Intention to Adopt Hearing Aids) was analyzed using *t* tests and Bayesian statistics as before. All results are summarized in Table 2. No significant age-related effects emerged for any of the variables examined (all *p*s > 0.21).

**TABLE 2.** Questionnaires investigating strategical aspects—NH young and NH older

	Groups Means		Statistics	
Strategies	NH Young	NH Older	*t* Test	BF
Coping strategies for listening challenges				
Negative/maladaptive	2.37 ± 0.56	2.28 ± 0.57	t(50) = 0.53, n.s., *d* = 0.15,	BF*01 = 3.20*
Positive/adaptive—verbal	3.03 ± 0.66	2.96 ± 0.63	t(50) = 0.39, n.s., *d* = 0.11,	BF*01 = 3.37*
Positive/adaptive—nonverbal	3.07 ± 0.86	3.35 ± 0.77	t(50) = 1.26, n.s., *d* = 0.35,	BF*01 = 1.89*
Coping strategies for hearing loss				
Positive/adaptive	3.92 ± 1.11	3.71 ± 1.12	t(50) = 0.68, n.s., *d* = 0.19,	BF*01 = 2.97*
Negative/maladaptive	1.72 ± 0.87	1.45 ± 0.72	t(50) = 1.22, n.s., *d* = 0.34,	BF*01 = 1.96*
Hearing aids				
Attitude toward hearing aids	6.39 ± 1.36	6.58 ± 0.76	t(50) = 0.63, n.s., *d* = 0.18	BF*01 = 3.05*
Behavioral intention to adopt hearing aids	4.35 ± 0.80	4.35 ± 0.94	t(50) = 0.00, n.s., *d* = 0.00	BF*01 = 3.59*

Mean, SD, and statistics (*t* tests) of behavioral strategies and attitude toward hearing aids and behavioral intention to adopt hearing aid values.

BF, Bayes factors; NH, normal hearing. Statistics Significant are highlighted in italics.

### The Role of HL in Older Adults on Metalistening

After confirming that age alone did not negatively affect any of the measured outcomes, the analyses focused on the effect of HL in older adults. As expected, older adults with NH and older adults with HL differed in hearing thresholds, calculated as the average of thresholds at 500, 1000, 2000, and 4000 Hz (NH older: 15.0 ± 3.9, range: 8.1 to 20.6; HL older: 29.1 ± 6.6, range: 21.3–48.1; *t*(51) = 9.38, *p* < 0.001, BF_10_ = 4.56). The HL older group was also slightly older, by approximately 3 yrs on average, than the NH older group (NH older: 64.6 ± 3.4, range: 60 to 76; HL older: 67.7 ± 5.2, range: 56 to 77; *t*(51) = 2.55, *p* = 0.01, BF_10_ = 2.30 × 10^9^).

#### Hearing in Noise Task: Performance, Confidence, and Metacognitive Monitoring

**Fig. 3.**
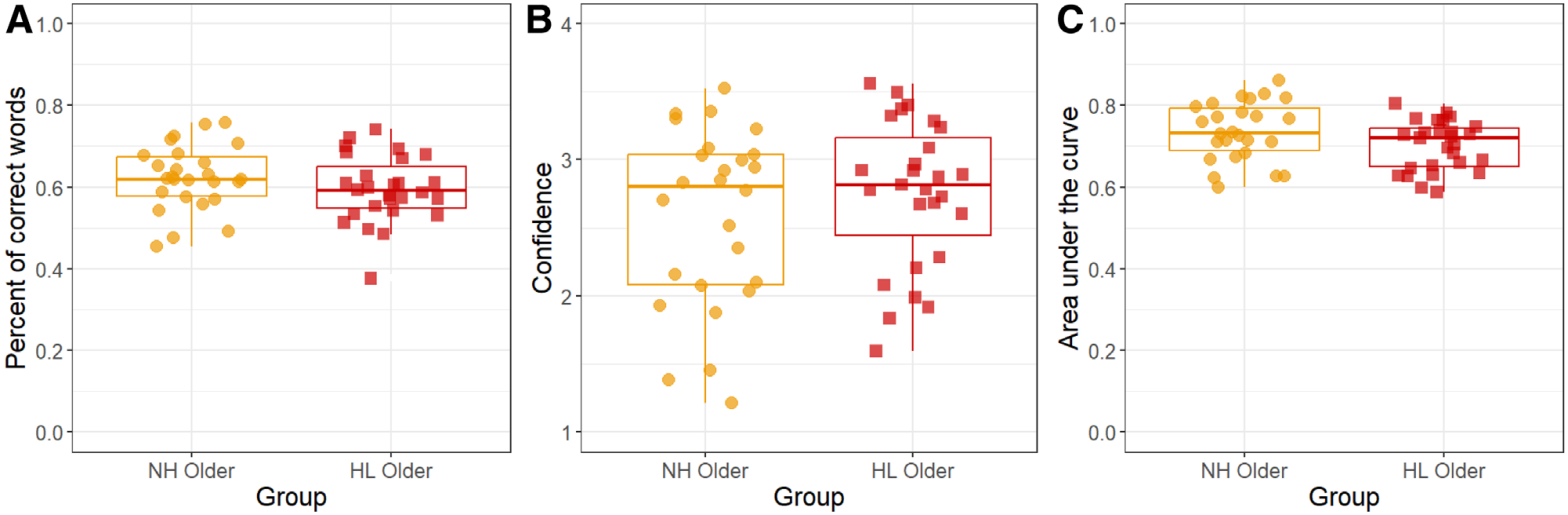
Hearing in noise task and metacognitive monitoring—NH older and HL older. Upper panel: hearing-in-noise task. (A) mean performance (percent correct)—scored as percentage of correctly reported words, (B) mean confidence, and (C) mean AUC values for the two groups: older adults with NH (circles) and older adults with hearing loss (squares). Values are reported in Supplementary Table 5, Supplemental Digital Content, https://links.lww.com/EANDH/B845. AUC indicates area under the curve; HL, hearing loss; NH, normal hearing.

As in The Role of Age on Metalistening, the analysis confirmed that both groups achieved comparable performance in the hearing in noise task, which was set to reach approximately 0.6 proportion correct (NH older: 0.62 ± 0.08; HL older: 0.60 ± 0.08, *t*(51) = 1.25, *p* = 0.22, *d* = 0.35, BF01 =1.83). The two groups also proved comparable in terms of confidence (NH older: 2.52 ± 0.63; HL older: 2.57 ± 0.65, *t*(51) = 0.28, *p* = 0.78, *d* = 0.08, BF01 =3.42) and metacognitive monitoring (NH older: 0.74 ± 0.07; HL older: 0.70 ± 0.06, *t*(51) = 1.94, *p* = 0.06, *d* = 0.53, BF01 = 1.53) (Fig. 3A).

Again, the correlation between percent corrects and confidence confirmed that, in both groups, participants who performed better in terms of accuracy were also those who reported higher confidence levels (NH older: *r* = 0.61, *p* < 0.001; HL older: *r* = 0.47, *p* = 0.01). For completeness, we also examined the effect of word order (see Supplementary Results, Supplemental Digital Content, https://links.lww.com/EANDH/B845).

#### Questionnaires About Metacognitive Aspects

The effect of HL on the LiCAS subscales and metacognitive knowledge was assessed using parametric tests and Bayesian statistics, reported in Table 3. A significant group difference emerged for LoC-HLL, with HL older showing higher scores than normal-hearing older adults (*t*(51) = 3.88, *p* < 0.001, *d* = 1.07). As anticipated in Questionnaire About Metacognitive Aspects, the LoC-HLL subscale includes items with and without explicit reference to hearing difficulties. Hence, when administered to adults with an actual auditory impairment, a comparison between the groups that takes into account this difference between items is more informative. Indeed, while a significant group difference was observed for the items with explicit reference to hearing difficulties (e.g., “When I don’t understand something, I tend to think I have a hearing problem”; *t*(29.36) = 4.14, *p* = 0.004), no such difference emerged for the more general items (e.g., “No matter how much I want it, I can’t understand well”; *t*(51) = 1.74, *p* = 0.09).

**TABLE 3.** Questionnaires about metacognitive aspects—NH older and HL older

	Means		Statistics	
Indices	NH Older	HL Older	*t* Test	BF
LiCAS-LoC				
HLL (LoC-HLL)	**2.99** ± **1.77**	**5.13** ± **2.21**	**t(51) = 3.88, *p* <.001, *d* = 1.07**	**BF**_***10***_ ***= 87.05***
CHL (LoC-CHL)	5.60 ± 2.65	5.04 ± 2.35	t(51) = 0.82, n.s., *d* = 0.23	BF_*10*_ *= 0.52*
LiCAS-SE				
Easy (SE-easy)	9.52 ± 0.73	9.06 ± 0.92	t(51) = 2.00 *p* = 0.05, *d* = 0.55*	BF_*10*_ *= 1.28*
Hard (SE-hard)	**8.20** ± **1.21**	**7.06** ± **1.69**	**t(51) = 2.81, *p* = 0.01, *d* = 0.77**	**BF**_***10***_ ***= 9.12***
Mean	**8.76** ± **0.85**	**7.99** ± **1.14**	**t(51) = 2.77, *p* = 0.01, *d* = 0.76**	**BF**_***10***_ ***= 8.81***
Metacognitive knowledge				
Easy	2.76 ± 1.38	3.35 ± 1.45	t(51) = 1.50, n.s., *d* = 0.41	BF_*10*_ *= 0.43*
Hard	5.31 ± 1.84	5.86 ± 1.72	t(51) = 1.13, n.s., *d* = 0.31	BF_*10*_ *= 0.39*

Mean, SD, and statistics (*t* tests) of LiCAS (SE, LoC), and metacognitive knowledge.

* *t* Test that did not remain significant when corrected for multiple comparisons.

BF, Bayes factors; CHL, control over hearing loss; HL, hearing loss; HLL, hearing loss likelihood; LiCAS, Listening Challenges Attitude Scale; LoC, locus of control; NH, normal hearing; SE, self-efficacy. Significant results are highlighted in bold.

Moreover, participants with HL showed lower general SE compared with those without hearing deficits (all *ps* < 0.05). As reported in Table 3, this difference was particularly evident for the SE-hard subscale (*t*(51) = 2.81, *p* = 0.01, *d* = 0.77), whereas the effect on the SE-easy subscale did not remain significant after correction for multiple comparisons.

As in Hearing in Noise Task: Performance, Confidence and Metacognitive Monitoring, the relationships between SE and LoC were examined, hypothesizing a directional link between these two variables (Cuevas et al. 2019). To test this, a regression analysis was conducted with LoC-HLL as the predictor and the SE mean index as the dependent variable, including group (NH and HL older) as an interacting factor. The results again revealed a significant effect of LoC-HLL (estimate = −0.28, *t* = −3.58, *p* < 0.001), suggesting that a more external LoC is associated with lower SE across both groups. No main effect or interaction involving group was observed (all *p*s > 0.78).

#### Behavioral Strategies

The effect of HL on behavioral strategies was examined using a series of *t* tests and Bayesian statistics. Results are summarized in Table 4. Significant differences emerged in the nonverbal strategies subscale, with HL older reporting less frequent use compared with NH older (*t*(51) = 2.13, *p* = 0.04, *d* = 0.59). However, it should be noted that this effect does not persist after correction for multiple comparisons. In addition, the adaptive deficit strategies subscale showed reduced use in the HL older group (*t*(51) = 3.01, *p* = 0.004, *d* = 0.83) and this effect remains significant after correction for multiple comparisons.

**TABLE 4.** Questionnaires investigating strategical aspects—NH older and HL older

	Means		Statistics	
Strategies	NH Older	HL Older	*t* Test	BF
Coping strategies for listening challenges				
Negative/maladaptive	2.28 ± 0.57	2.01 ± 0.55	t(51) = 1.79, n.s., *d* = 0.49	BF_*10*_ *= 1.61*
Positive/adaptive-verbal	2.96 ± 0.63	3.08 ± 0.84	t(51) = 0.61, n.s., *d* = 0.17	BF_*10*_ *= 0.40*
Positive/adaptive-nonverbal	**3.35** ± **0.77**	**2.74** ± **1.25**	**t(Welch**)(51) = 2.13, *p* = 0.04, *d* = 0.59*	**BF**_***10***_ ***= 2.61***
Coping strategies for hearing loss				
Positive/adaptive	**3.71** ± **1.12**	**2.59** ± **1.54**	**t(Welch**)(51) **= 3.01, *p* = 0.004, *d* = 0.83**	**BF**_***10***_ ***= 5.16***
Negative/maladaptive	1.45 ± 0.72	1.20 ± 0.50	t(51) = 1.48, n.s., *d* = 0.28	BF_*10*_ *= 0.69*
Hearing aids				
Suitable adjective	6.58 ± 0.76	6.37 ± 0.93	t(51) = 0.89, n.s., *d* = 0.24	BF_*10*_ *= 0.32*
Behavioral intention	4.35 ± 0.94	4.12 ± 0.91	t(51) = 0.90, n.s., *d* = 0.25	BF_*10*_ *= 0.45*

Mean, SD, and statistics (*t* tests) of behavioral strategies and attitude toward hearing aids and behavioral intention to adopt hearing aid values.

* *t* Test that did not remain significant when corrected for multiple comparisons.

BF, Bayes factors; HL, hearing loss; NH, normal hearing.

These results, together with those in Questionnaires About Metacognitive Aspects, indicate less frequent use of nonverbal and adaptive HL strategies in HL older individuals, alongside lower SE and a more external LoC, for items with explicit reference to HL. Given that reduced strategy use may have important functional consequences and considering evidence suggesting a link between metacognitive factors and strategy implementation (Amran et al. 2021), a series of regression analyses was performed to investigate whether LoC and SE predicted the use of nonverbal and hearing deficit strategies, taking group into account.

Considering LoC-HLL as a predictor revealed no significant effects on either nonverbal or deficit strategies (all *ps* > 0.05), nor any interaction with group (all *ps* > 0.07). The impact of perceived control over HL (LoC-CHL), which reflect assesses the participant’s perceived ability to improve hearing issues, was also investigated. For nonverbal strategies, LoC-CHL significantly predicted strategy use, with individuals reporting a more internal LoC (higher values) being more likely to implement non-verbal strategies (estimate = 0.24, *t* = 2.92, *p* = 0.005). This effect was particularly pronounced in the HL older group (trends = 0.24) compared with the NH older group (trends = 0.01, estimate = 1.77, *t* = 2.09, *p* = 0.04), possibly reflecting greater variability in strategy frequency within the HL group.

When SE was examined as a predictor, a main effect on nonverbal strategies was observed: lower SE was associated with greater use of nonverbal strategies, independent of group (estimate = −0.39, *t* = −2.21, *p* = 0.03; other *ps* > 0.12).

For further analysis studying the relationship between metacognition and strategies, considering the entire sample, we refer to the Supplementary Materials, Supplemental Digital Content, https://links.lww.com/EANDH/B845. These exploratory results suggest that individuals with higher LoC-CHL values tended to implement verbal strategies more frequently.

#### Correlations With Hearing Experience and Age

To further examine the influence of age and HL, we conducted Spearman correlation analyses. While The Role of Hearing Loss in Older Adults on Metalistening examined HL as a categorical variable (HL versus NH), this section investigates HL as a continuous variable across both older adult groups. Spearman correlation analyses were conducted, including variables identified as significant in previous analyses (see Questionnaires About Metacognitive Aspects and Behavioral Strategies), alongside age and PTA thresholds and speech perception thresholds. Results showed that age correlated with both tonal and vocal audiometric measures. More importantly, self-beliefs and coping strategies related to HL were significantly correlated with PTA thresholds and, to a lesser extent, with speech perception thresholds, but not with age (Table 5, Supplementary Figures 2 and 3, Supplemental Digital Content, https://links.lww.com/EANDH/B845). These findings support the interpretation that differences observed between groups are primarily attributable to auditory experience rather than age.

**TABLE 5.** Correlations with hearing experience and age

Variables	Age	Hearing Threshold	Perception Threshold 50%
Age	-		
Hearing threshold (tonal audiometry)	0.39**	-	
Perception threshold 50% (vocal audiometry)	0.36*	0.76***	-
Hearing loss likelihood	0.07	0.48***	0.49***
Self-efficacy easy	−0.14	−0.30*	−0.13
Self-efficacy difficult	−0.24	−0.35*	−0.23
Self-efficacy mean	−0.19	−0.36**	−0.27*
Coping strategies for listening challenges (positive/adaptive-nonverbal)	−0.08	−0.18	−0.08
Coping strategies for hearing loss (positive/adaptive)	0.06	−0.32*	−0.42**

Spearman correlation between age hearing threshold (tonal audiometry), perception threshold 50% (vocal audiometry), hearing loss likelihood, self-efficacy easy, self-efficacy difficult, self-efficacy mean, coping strategies for listening challenges (positive/adaptive-nonverbal), coping strategies for hearing loss (positive/adaptive).

* P < .05,

** p < .01,

*** p < .001.

## DISCUSSION

### The Role of Age and HL on Metalistening

This study examined the contributions of age and HL to metalistening. With respect to the role of age, the present findings provide further evidence that age per se does not impair metalistening. When metacognitive monitoring was measured using the hearing-in-noise task, no difference as a function of age emerged. If anything, a slight advantage for older compared with younger adults became apparent when we doubled sample size by pooling the current results with those obtained by Giovanelli et al. (2024). The decision to include data from this previous work was motivated by the full methodological overlap between studies and the opportunity to assess our findings under the lenses of increased statistical power. Consistently, the results of the present study also showed no differences between younger and older adults in terms of SE, LoC, and metacognitive knowledge, as assessed through standardized questionnaires.

This absence of age-related effects on metalistening for NH adults aligns with previous research using different experimental paradigms. Valzolgher et al. (2024) compared younger and older adults by asking them to evaluate the effort required and their confidence while listening to a continuous story presented with fluctuating background noise. This design enabled the authors to derive a novel measure of metacognitive monitoring, termed metacognitive tracking. Both groups adjusted their online judgments of effort and confidence in line with variations in the SNR, suggesting that aging does not impair individuals’ capacity for continuous online evaluation of internal states such as effort and confidence. Similarly, Giovanelli et al. (2025) investigated age effects on the metacognitive awareness of lipreading, an adaptive strategy that facilitates face-to-face communication in noisy environments. The comparison of gain assessments by Giovanelli et al. between younger and older adults revealed comparable responses across groups, further supporting evidence of preserved metacognitive abilities in listening contexts. Older adults effectively exploited lipreading to enhance their listening experience and recognized the associated benefits for speech perception, indicating intact metacognitive awareness of strategy effectiveness.

With respect to the role of HL on metalistening the comparison between older adults with NH and older adults with HL yielded no significant difference for metacognitive monitoring in the hearing-in-noise task. Yet, differences between older adults with and without HL emerged in self-report measures of metalistening. Specifically, older adults with HL reported higher scores on the LoC-HLL scale in the items that explicitly refer to hearing difficulties. When considered in the context of individuals with HL, these items indicate acknowledgement and awareness of hearing difficulties. In addition, they reported lower SE compared with their normal-hearing peers. Taken together, these findings suggest that older adults are generally able to monitor their listening challenges and are aware of their hearing-related limitations when experiencing HL. Yet, they may be undermining in their SE by the presence of HL.

These results are consistent with previous findings by Dammeyer et al. (2018), who reported that young individuals with HL, particularly young individuals with HL, particularly those without assisted hearing, exhibited lower SE compared with their normal-hearing peers. The authors interpreted this difference as reflecting the communicative and social challenges typically faced by individuals with hearing impairment. Similarly, in a study examining the relationship between hearing status and SE in adults, Dachman (Reference Note 1) found that individuals with HL reported lower levels of SE. Overall, the acknowledgement and awareness of auditory difficulties and the reduced SE in individuals with HL aligns with the findings from our hearing-in-noise task that document preserved monitoring and metacognitive awareness in this population.

The present data suggest that metacognitive monitoring of listening is overall preserved in both older adults with NH and those with HL. It is important to note that the assessment used a task designed to control for differences in cognitive functions such as working memory and attention, which are known to vary between younger and older populations. This methodological choice likely minimized confounding effects that could obscure true metacognitive abilities. However, it remains plausible that alternative tasks, which do not regulate for these cognitive variables, might reveal metacognitive deficits linked to broader cognitive demands. Indeed, previous research investigating metacognition in more cognitively demanding listening contexts, such as false hearing tasks (Rogers et al. 2012), has reported impairments in older adults (e.g., reduced metacognitive accuracy), suggesting that when working memory load is elevated, differences in metacognitive monitoring become more apparent. This interpretation aligns with the ELU model proposed by Rönnberg et al. (2008), which emphasizes the critical role of working memory in supporting language comprehension under challenging conditions. Moreover, studies in other cognitive domains have shown that expected task difficulty modulates metacognitive confidence and monitoring, further supporting a link between working memory and metacognition (Sherman & Seth 2021). Therefore, while perceptual metalistening per se appears intact among older adults regardless of hearing status, more complex, ecologically valid listening scenarios involving substantial working memory engagement may expose age- and hearing-related declines in metacognitive monitoring.

### Influence of Hearing Status on Behavioral Strategies in Older Adults

This study also investigated the impact of age and HL on the ability to manage hearing challenges and implement compensatory strategies.

The reported use of strategies for managing challenging listening situations was unaffected by age, as suggested by the lack of differences between strategic assessment of older and younger adults with NH. However, preliminary evidence for an impact of hearing status emerged, with older adults with HL reporting less frequent use of such strategies compared with their normal-hearing peers. Item-level analyses revealed that this difference was most evident for strategies involving the use of visual cues, such as focusing on the speaker’s face during communication. Although individuals with HL might be expected to rely more on visual strategies, given the potential benefit of visual cues in supporting speech perception (Winneke & Phillips 2011), findings by Giovanelli et al. (2025) offer a possible explanation. In their study, both younger and older adults recognized the benefits of lipreading. However, older adults (particularly those less skilled in lipreading) reported a smaller perceived reduction in effort when visual cues improved. This suggests that, for older adults, accessing visual information may increase cognitive demands, potentially reducing the perceived benefit of such strategies. In this perspective, HL may further elevate the cognitive load required for effective listening, influencing strategic choices in challenging situations. The tendency to adopt or avoid certain strategies may thus reflect a cost–benefit evaluation shaped by cognitive load (Touron 2015), potentially leading to maladaptive behaviors.

A similar pattern emerged for strategies aimed at managing HL. The present study showed that older adults with NH were more likely than those with HL to report the intention to inform others about potential hearing difficulties when facing challenging listening situations. More specifically, informing others implies being exposed to judgements, which might discourage such verbal strategies. Notably, in the present study, older adults with NH responded by imagining a hypothetical behavior, whereas those with HL were asked to reflect on their actual behavior, which, at least in principle, should already be part of their daily experience. It is plausible that individuals without HL do not anticipate the emotional impact of shame or stigma when considering a hypothetical scenario, while such factors may play a significant role in shaping the behavior of individuals who experience HL in real life (see, for a review.

Finally, attitudes toward hearing aids were examined by assessing participants’ perceptions of hearing aid usefulness and their behavioral intention to adopt them (Adorni et al. 2022). Previous research has shown that the wording used by significant others, such as caregivers or healthcare professionals, can positively influence hearing aid acceptance. For example, presenting HL in more “medical/technical” versus more “everyday” terms has been found to affect attitudes toward hearing aids (Adorni et al. 2022). In the present study, we aimed to explore whether attitudes and intentions toward hearing aids, considered as a form of adaptive coping strategy, were associated with individual differences in metacognitive abilities. The analyses revealed no differences between participants with and without HL and no significant associations between metacognitive measures and hearing aid attitudes. Nonetheless, in the present study, hearing aids were rated as useful (with mean scores around 6.4 of 7) and the intention to adopt them in case of hearing difficulties was high (averaging 4.2 of 5). Our findings suggest a positive attitude toward hearing aids among older adults, regardless of hearing status. However, the present data did not allow for disentangling the two alternative hypotheses outlined in the introduction, namely, whether favorable attitudes toward hearing aids reflect a metacognitively driven adaptive strategy or represent a last-resort option considered only when other compensatory strategies fail.

### Exploring the Relationship Between Metacognitive Dimension and Reported Use of Listening Strategies

The relationship between metacognitive dimensions and the reported use of listening strategies was examined through an exploratory analysis. A significant association emerged between SE and LoC. Previous research has identified LoC as a predictor of SE (Cuevas et al. 2019), and the present findings extend this relationship to the domain of listening, which remains largely underexplored. Specifically, a more external LoC consistently predicted lower SE across groups, corroborating the conceptual link between these constructs. This evidence also aligns with the perspective proposed in a recent study introducing the LiCAS tool, which sustain the inter-relation of such constructs in shaping the listening experience, proposing a tool that jointly assesses these dimensions within the context of listening in adverse conditions (Giovanelli et al., Reference Note 2).

Notably, lower SE was associated with a greater use of nonverbal strategies, irrespective of group. Although this pattern might appear counterintuitive, given that higher SE is generally expected to foster proactive engagement in effective behaviors (Pajares & Usher 2008; Cook & Artino 2016; Koponen et al. 2021), it may reflect behavioral tendencies linked to the subjective experience of hearing difficulties. Individuals with lower SE might favor compensatory strategies that do not require explicitly revealing their challenges, such as relying more heavily on visual cues or adjusting their position within the listening environment.

Findings also suggested that a more internal LoC, particularly in the LoC-CHL scale, was associated with a higher frequency of both verbal and nonverbal strategy use. This association was especially evident within the group with HL, possibly reflecting greater variability in strategic behaviors among individuals with HL. These results support the hypothesis that an internal sense of control promotes adaptive behavioral responses when facing cognitive or perceptual challenges. Moreover, they underscore the potential role of fostering internal control beliefs in supporting the implementation of effective strategies, particularly for older adults coping with HL.

The observed associations between metacognitive beliefs, monitoring, and compensatory behaviors can be interpreted within the classical framework proposed by Nelson and Narens (1990), which describes metacognition as the interplay between monitoring and control processes. Monitoring provides information about performance (e.g., perceived success, effort, or confidence), while control mechanisms use this information to guide subsequent behavior.

In this light, our findings seem to suggest that changes in SE and a reduced perception of control over listening challenges may influence the effective translation of monitoring outcomes into adaptive behavioral adjustments. This interpretation aligns with models by Koriat and Goldsmith (1994, 1996) and Koriat et al. (2006), which highlight the role of metacognitive judgments in regulating effort and decision-making and it is in line with the importance of considering nonaudiological factors in the studying of predictors of coping behaviors (Gomez & Madey 2001). From this perspective, the decision to engage or not to engage in compensatory actions when facing auditory challenges may reflect a form of metacognitive control grounded in confidence and control beliefs. Overall, these findings support a view of metalistening as not only reflecting awareness of listening success but also contributing to behavioral regulation in everyday communication.

### Limitations, Future Research Direction, and Clinical Implication

#### Task Design and Experimental Paradigm

This study assessed metacognitive monitoring using a hearing-in-noise task specifically designed to minimize variability in attention, working memory, and auditory abilities between groups, thereby ensuring comparable performance levels. While this methodological choice strengthened internal validity, it may have reduced the task’s ecological complexity, resulting in a relatively static listening challenge. Modulating the SNR (see, for instance, Valzolgher et al. 2024) or introducing varying degrees of task difficulty could make the paradigm more dynamic and ecologically valid. Future studies should explore whether older adults maintain accurate metacognitive monitoring under increasingly adverse or unfamiliar conditions.

#### Self-Report Measures and Strategy Assessment

The reliance on self-report questionnaires to assess metacognitive beliefs and strategy use represents another limitation, as these tools may be subject to social desirability biases or self-perception inaccuracies. To complement self-report data, future studies should use experimental or observational methods, such as simulated interactive environments, to capture participants’ actual behavior in ecologically valid listening situations (Keidser et al. 2020). This approach would offer deeper insight into how metacognitive awareness translates into real-world strategic behavior.

#### Bridging Awareness and Behavior

Although this study contributes to understanding the link between metacognition and behavioral responses to listening challenges, further research is needed to clarify how metacognitive awareness, beliefs, and cognitive demands interact in shaping adaptive and maladaptive behaviors (Hess et al. 2016). A critical next step is to investigate the conditions under which metacognitive awareness effectively promotes strategy use, helping to bridge the gap between knowing and doing in the management of adverse listening situations. Future research could, for instance, incorporate additional variables beyond those already considered (such as perceived strategy effectiveness, individuals’ perceptions of the effectiveness of coping strategies in managing HL in daily life, adjustment to HL, and social support, see Gomez & Madey 2001) by integrating metacognitive aspects into the analysis of predictors of coping strategies in the context of hearing in noise.

#### Clinical Implications and Practical Considerations

Beyond its theoretical relevance, this study raises important considerations for clinical practice. Incorporating metacognitive assessments into audiological care may help professionals gain a deeper understanding of patients’ listening experiences and perceptions of control in challenging situations and thus optimizing person-centered are for older adults (Kestens et al. 2025). Addressing these aspects through targeted counseling or training could enhance both the use of communication strategies and the acceptance of interventions such as hearing aids (McCormack & Fortnum 2013). Furthermore, while previous studies have investigated a range of factors influencing hearing aid adoption, even beyond the audiological factors (Meyer & Hickson 2012; Hickson et al. 2014), future research could also consider the role of metacognitive variables that may foster a more favorable disposition toward hearing aid use.

## CONCLUSION

The present findings offer initial insight into how age and HL affect metalistening, a largely underexplored area with important implications for strategic listening. While older adults with and without HL showed similar awareness of listening difficulties, those with HL reported lower SE and used adaptive strategies less often. This suggests that beliefs about one’s ability to manage challenges may influence strategic behavior. Further research should explore how cognitive load, stigma, and individual attitudes shape listening strategies, to better support older adults, particularly those with HL, through targeted interventions.

## ACKNOWLEDGMENTS

The authors thank all participants who took part in this experiment and Chiara Visentin for helping to test the device audio settings.

## Supplementary Material
