# Supplementary material for: Metacognition for Listening in Noise: The Role of Age and Hearing Loss
Source: Ear Hear. 2026 Mar 9;47(4):1045–58. doi: 10.1097/AUD.0000000000001798 (PMC13252978; doi:10.1097/AUD.0000000000001798)
Supplement: Supplementary file 1 [file aud-47-1045-s001.pdf]

Supplementary Materials

METACOGNITION FOR LISTENING IN NOISE: THE ROLE OF AGE AND HEARING LOSS

1. Supplementary methods

Receiver Operator Characteristic (ROC) Analysis

As in Giovanelli et al., (2023), metacognitive monitoring was measured as the relation between actual percent correct and the corresponding confidence judgement in any given trial. Every selected word was categorised as either correct or incorrect, while confidence scores ranged from 1 to 4. To assess each participant's metacognitive-monitoring efficiency, we employed Receiver Operator Characteristic (ROC) Analysis. This analysis considers "good monitoring" to be those trials in which a correct answer is associated with a high-confidence score (HIT) or, equally, those trials in which low performance (percent correct) were associated with low confidence (CORRECT REJECTION). Conversely, trials characterised as "poor monitoring" are those having incorrect responses associated with high-confidence scores (FALSE ALARMS) or having correct responses associated with low confidence (MISS). Note that the ROC analysis considers only HIT and FALSE ALARM in determining the quality of metacognitive monitoring. This non-parametric analysis examines all potential criteria for dividing confidence into high or low categories. For each criterion, a matrix is generated to count the various types of responses obtained. If we represent the HIT rate and FALSE ALARM rate obtained for each criterion on a graph, we obtain the ROC curve (see Fleming & Lau, 2014). The value of the Area Under the Curve (AUC) represents the metacognitive accuracy of the participant. AUC values of 0.5 correspond to a monitoring ability at chance level. Higher AUC values correspond to greater metacognitive efficiency. We computed a ROC curve (and its AUC) for each word position. The overall metacognitive monitoring of a participant is the mean AUC of the AUC values for each word position. Analyses of metacognitive monitoring were carried out using an ad-hoc MATLAB script.

2. Supplementary Results

Hearing in noise task

Age and word order

Because Giovanelli et al. (2023) found differences between groups as a function of word order in the sentence, three follow-up analyses with group and word order (1st, 2nd, 3th or 4th) as independent variables were conducted. When considering the percentage of correct responses as dependent variable, a main effect of word order emerged. Consistent with Giovanelli et al. (2022), last words in the sentence were recognized better than initial ones ($F(3,150) = 233.25$, $p < 0.001$, $\eta^2 = 0.69$). A significant interaction effect between group and word order also emerged ($F(3,150) = 3.99$, $p = 0.009$, $\eta^2 = 0.01$), suggesting that older NH recognized the first two words better than young NH (simple main effect for the first two words: $ps < 0.01$, for the third and fourth: $ps > 0.35$). This interaction was unexpected and we deem its discussion beyond the scopes of this study (see Figure 2D). When considering confidence, a main effect of word order also reached significance. This suggests that participants were more confident when asked to rate the first two words of the sentences as compared to the third and the fourth word (F (2.57,128.44) = 54.95, $p < 0.001$, $\eta^2 = 0.20$; no other main effects were found: $ps > 0.21$) (see Figure 2E). When considering metacognitive monitoring, a main effect of word order emerged, caused by better monitoring for words in positions 3 and 4 for both age groups ($F(3,150) = 15.08$, $p < 0.001$, $\eta^2 = 0.13$; no main effects were found: $ps > 0.21$) (see Figure 2F) (Supplementary Table 3 for all means).

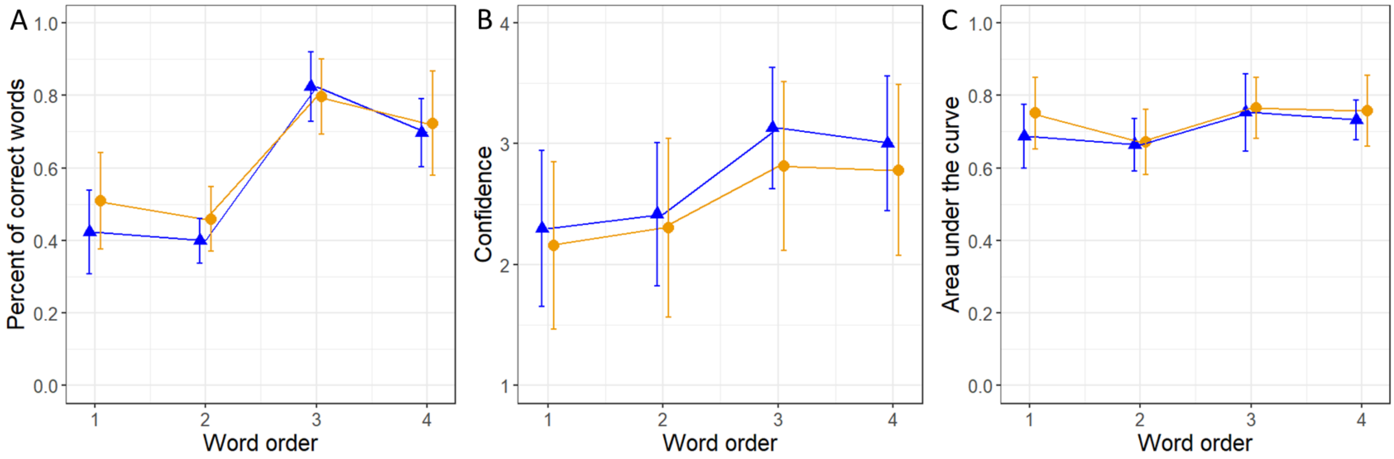

HEARING IN NOISE TASK AND METACOGNITIVE MONITORING – NH YOUNG AND NH OLDER. (A) Percent of correct words, (B) mean confidence and (C) metacognitive monitoring (AOC scores) values as a function of word order for the two groups: young (triangles) and older adults with normal hearing (circles) values for the two groups NH young (triangles) and NH older (circles). Values are reported in Supplementary Table 5.

Hearing loss and word order

The effect of word order was examined also as a function of the presence vs. absence of hearing loss in the older group. When considering the percentage of correct, a main effect of word order was observed. The last words in the sentence were recognized more frequently than the first ones (F (2.56, 130.67) = 93.46, $p < 0.001$, $\eta^2 = 0.50$, no other main effects were found: *ps* > 0.10). When considering confidence, a main effect of word order was also found, suggesting that participants were more confident when asked to rate the first two words of the sentences than the third and the fourth one (F (2.57, 131.13) = 34.92, $p < 0.001$, $\eta^2 = 0.12$; no other main effects were found: *ps* > 0.34). When considering metacognitive monitoring, a main effect of word order was found, suggesting that participants were more confident when asked to rate the first words of the sentences than the second part (F(3,150) = 17.48, $p < 0.001$, $\eta^2 = 0.13$; no main effects were found: *ps* > 0.06) (see Figure 3 and Supplementary Table for all means).

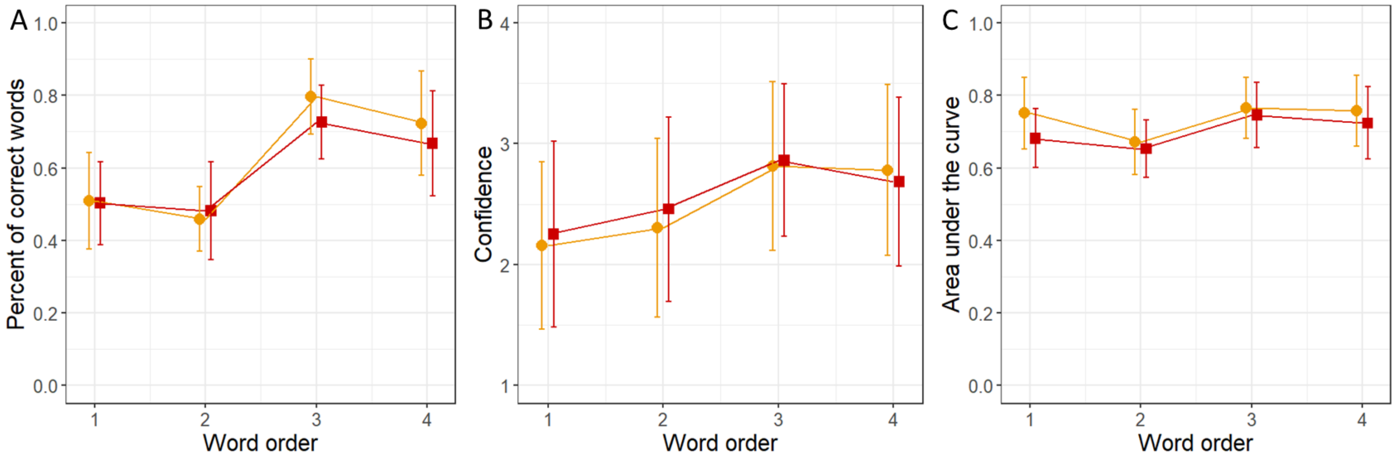

HEARING IN NOISE TASK AND METACOGNITIVE MONITORING – NH OLDER and HL OLDER. Hearing-in-noise task, studied as a function of word position in the sentence. (A) mean performance (percent correct), (B) mean confidence and (C) mean AUC (area under the curve) values for the two groups: NH older (circles) HL older (squares). Values are reported in Supplementary Table 5.

Considering the hearing in noise task, we observed some differences between the data in this study and those reported in the previous one by Giovanelli et al. (2023) we intended to replicate. We found no differences between young and older adults regarding performance or confidence, while Giovanelli and colleagues (2023) found higher performance and higher confidence in young as compared to older adults. Here, the adaptive procedure enabled both groups, despite individual variability, to reach comparable levels of performance (around 60% speech intelligibility). This improvement reduces the potential confound related to performance gaps observed in the previous study, allowing us to show that perceived confidence also did not differ between young and older adults. The confidence differences reported by Giovanelli et al. (2023) largely reflected group differences in performance. This interpretation is further supported by a supplementary analysis we run, which combined data from both experiments: pooling the two studies together resulted in a sample of 105 participants (56 young and 49 old adults). Note that this is possible because the methodological choices adopted for this task match in all respects those adopted by Giovanelli et al. (2023). Through regression analysis, we found that performance influenced confidence only in Giovanelli's original experiment, where group differences emerged, but not in ours (confidence ~ experiment * performance, estimate = 6.47, t = 3.04, p = 0.003). By re-analysing the data, we observed that the two groups also proved comparable for both performance (NH young: 0.61±0.08; NH older: 0.61±0.07, t(130) = 0.42, p = 0.78, d = 0.08, *BF01 = 4.47*) and confidence (NH young: 2.74±0.58; NH older:

2.61±0.64, *t*(130) = 1.11, *p* = 0.27, *d* = 0.22, *BF10 = 2.80*). However, it is worth noticing that while considering also word order in the analysis, we did observe a slight advantage in performance for young compared to older for certain words and there was an age effect on confidence, with younger individuals being more confident than older ones. This, however, could be a consequence of the slight performance advantage mentioned above. Notably, however, we found significantly better monitoring for older as compared to younger individuals (NH young: 0.70±0.05; NH older: 0.73±0.06, *t*(103) = 2.27, *p* = 0.03, *d* = 0.45, *BF01 = 2.01*). This finding is in line with the previous study, in which NH older showed better metacognitive monitoring on the last two words of sentences compared to NH young. These further analyses confirm that there is no negative effect of age on metacognitive monitoring, as measured by our task. If anything, a slight advantage for older adults can emerge.

Exploring the relationship between metacognition and behavior

These data allow us to explore, considering the entire sample, the relationship between metacognitive aspects (SE, LoC) and behavior (particularly focusing on adaptive behavior, such as verbal strategies, non-verbal strategies, adaptive coping strategies for listening challenges, and behavioral intentions related to hearing aids). We ran regressions to study when metacognitive aspects might predict these strategies. Thus, the strategies were entered as dependent variables and the metacognitive variables as independent variables, irrespectively of the group.

We only considered LoC CHL and not LoC HLL because it could have been too influenced by the fact that our sample included individuals with HL (see Discussion in the Manuscript).

Variables	SE	LoC CHL
**Coping strategies for listening challenges**		
Positive/Adaptive - Verbal	t = -0.49, p = 0.62	t = 1.46, p = 0.15
Positive/Adaptive - Non-verbal	t = -0.50, p = 0.62	**t = 3.38, p = 0.001**
**Coping strategies for hearing loss**		
Positive/Adaptive	t = 0.15, p = 0.88	t =1.78, p = 0.08
**Hearing aids**		
Behavioural intention	**t = 2.57, p = 0.01***	t = 0.65, p =0.42

** indicates t-test that did not remain significant when corrected for multiple comparisons*

People with higher values of LoC (Locus of Control) CHL (Chronic Health Locus) were the ones who implemented more verbal strategies. Having an internal locus of control allows individuals to take more action, as we expected. People with higher SE (Self-Efficacy) scores (meaning they reported less effort) were also more likely to use hearing aids. This could be related to the fact that those who exert less effort are likely individuals who have not yet been influenced by stereotypes or factors that might discourage them from considering hearing aids.

3. Supplementary Figures

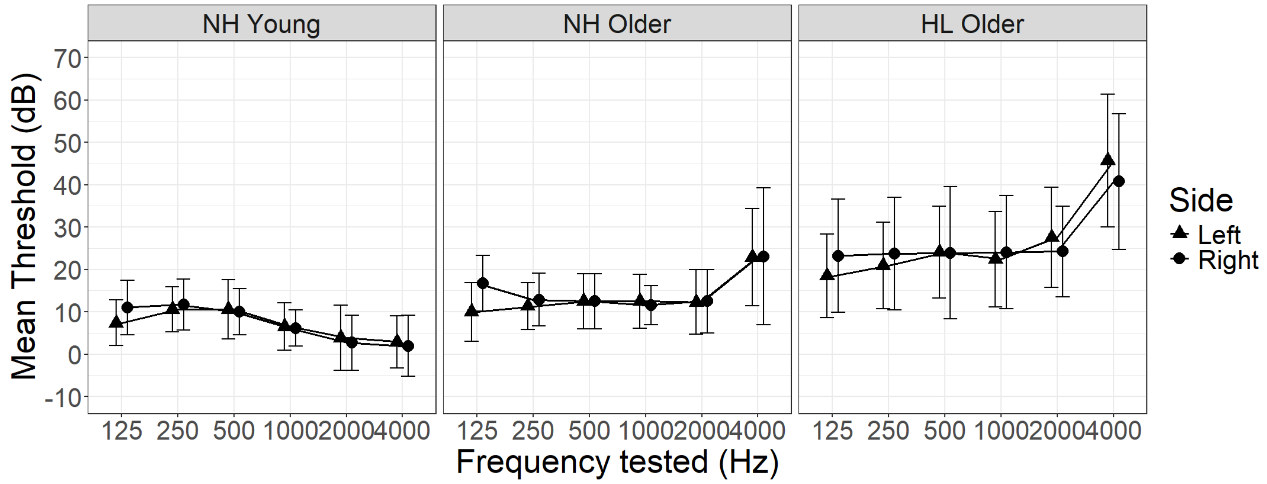

Supplementary Figure 1. HEARING THRESHOLD. Average hearing thresholds in decibels (dB) for each tested frequency in both ears across the three age groups (NH young, NH older, HL older) and as a function of side (left, right).

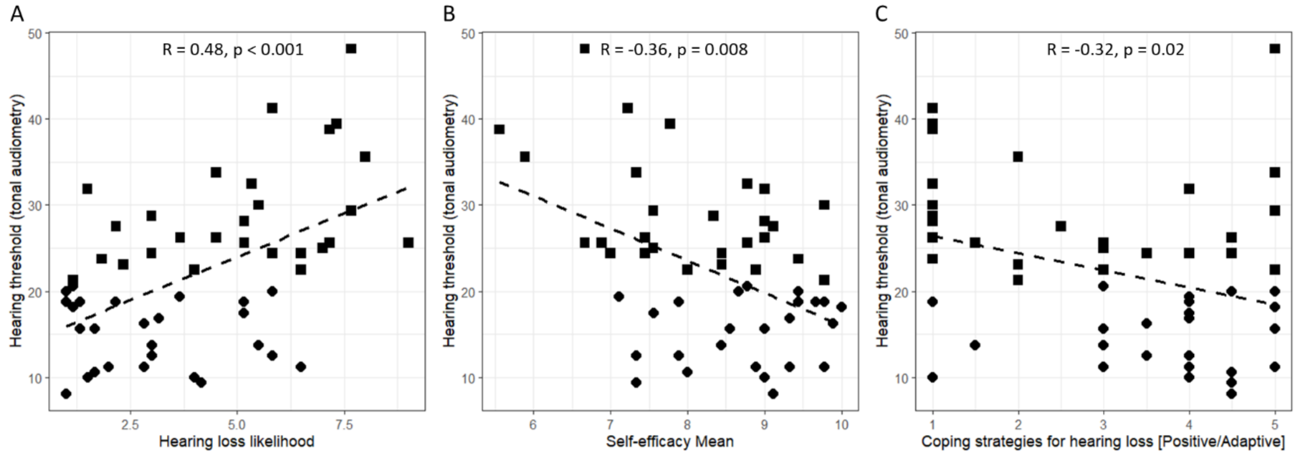

Supplementary Figure 2. Spearman correlation between Age Hearing threshold (tonal audiometry) and Hearing loss likelihood (A), Self-efficacy Mean (B), Coping strategies for hearing loss [Positive/Adaptive] (C). Individual data points for each participant are shown: older adults with normal hearing (circles) and older adults with hearing impairments (squares).

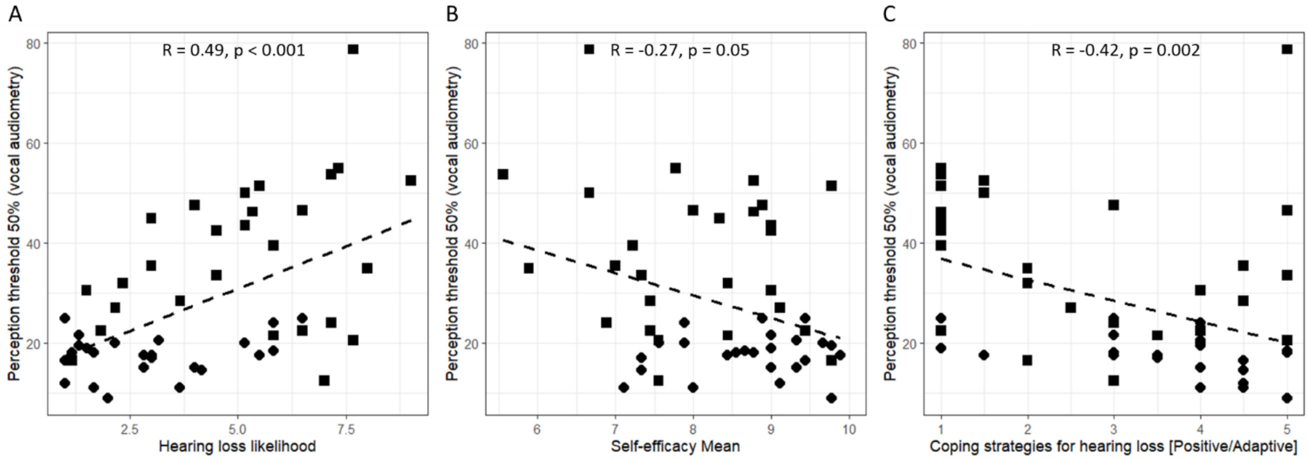

Supplementary Figure 3. Spearman correlation between Vocal Audiometry and Hearing loss likelihood (A), Self-efficacy Mean (B), Coping strategies for hearing loss [Positive/Adaptive] (C). Individual data points for each participant are shown: older adults with normal hearing (circles) and older adults with hearing impairments (squares).

4. Supplementary Tables

ID	*Age*	*Group*	*Mean Threshold left ear*	*Mean Threshold right ear*	*50% (left ear)*	*50% (right ear)*	*MoCA score*	*Years of education*	*Presence of Tinnitus*
1	23	Young	10	7,5	12	11	-	17	No
2	68	HL - Older	45	26,25	47	23	29	17	No
3	19	Young	3,75	0	4	7	-	14	No
4	68	HL - Older	26,25	25	52,5	47,5	29	18	Yes
5	65	NH - Older	12,5	10	30	20	27	13	Yes
6	20	Young	0	3,75	11	11	-	14	No
7	24	Young	1,25	1,25	8	5	-	18	No
8	21	Young	10	5	11	11	-	13	No
9	67	HL - Older	27,5	17,5	53	42	27	27	No
10	25	Young	8,75	3,75	11	12	-	18	No
11	20	Young	8,75	1,25	8	6	-	14	No
12	23	Young	5	6,25	10	9		18	No
13	20	Young	2,5	3,75	9	11	-	13	No
14	19	Young	10	10	11	7	-	13	No
15	19	Young	-1,25	2,5	8	7	-	13	No
16	20	Young	7,5	3,75	4	10	-	13	No
17	21	Young	5	6,25	9	13	-	15	No
18	19	Young	6,25	3,75	7	9	-	13	No
19	19	Young	1,25	8,75	7	6	-	13	No
20	21	Young	1,25	3,75	10	7	-	13	No
21	24	Young	16,25	16,25	15-20	18	-	17	No
22	20	Young	5	5	12	13	-	15	No
23	22	Young	11,25	12,5	10	12	-	15	No
24	62	NH - Older	7,5	8,75	16	8	30	12	No
25	63	NH - Older	18,75	17,5	-	-	29	12	No
26	69	NH - Older	13,75	23,75	25	25	26	19	No
27	67	HL - Older	36,25	15	60	45	29	14	No
28	60	HL - Older	23,75	25	22	21	28	22	No
29	22	Young	10	10	12	12	-	16	No
30	69	HL - Older	22,5	30	28	29	28	16	No
31	63	NH - Older	11,25	10	14	8	27	10	Yes
32	65	HL - Older	26,25	21,25	20	25	27	15	No
33	65	NH - Older	26,25	13,75	15	18	27	16	No
34	77	HL - Older	31,25	23,75	30	24	27	16	No
35	60	NH - Older	16,25	11,25	13	22	28	18	No
36	62	NH - Older	15	23,75	10	12	26	18	No
37	77	HL - Older	32,5	16,25	38	33	27	16	No
38	66	NH - Older	21,25	13,75	22	18	29	13	Yes
39	62	NH - Older	11,25	8,75	17	13	30	16	Yes
40	61	NH - Older	21,25	20	18	18	28	13	No
41	69	HL - Older	32,5	31,25	28	33	28	10	No
42	69	HL - Older	37,5	30	38	29	28	17	Yes
43	63	NH - Older	13,75	8,75	8	10	30	15	Yes
44	72	HL - Older	23,75	25	20	25	13	13	No
45	67	NH - Older	22,5	17,5	19	18	24	24	No
46	69	HL - Older	22,5	28,75	20	28	27	10	No
47	67	HL - Older	33,75	25	18	23	27	14	No
48	65	NH - Older	21,25	12,5	18	23	28	19	No
49	65	HL - Older	20	22,5	13	20	27	14	No
50	65	NH - Older	5	22,5	18	17	27	18	No
51	61	NH - Older	13,75	8,75	15	15	29	14	No
52	65	NH - Older	17,5	20	18	22	28	13	Yes
53	76	HL - Older	25	21,25	29	35	26	8	No
54	72	HL - Older	38,75	43,75	34	45	26	13	Yes
55	69	NH - Older	15	22,5	17	22	28	23	No
56	66	HL - Older	25	25	10	15	29	13	No
57	63	NH - Older	11,25	13,75	25	23	29	16	No
58	70	NH - Older	16,25	16,25	17	18	30	16	Yes
59	63	NH - Older	11,25	8,75	18	20	28	11	No
60	64	NH - Older	18,75	18,75	17	23	28	17	Yes
61	76	NH - Older	15	16,25	18	18	26	16	No

62	63	NH - Older	7,5	11,25	13	16	28	17	No
63	64	NH - Older	13,75	17,5	18	25	29	14	No
64	64	NH - Older	13,75	11,25	22	12	30	18	No
65	67	HL - Older	35	43,75	45	65	30	9	No
66	72	HL - Older	31,25	25	45	42	26	9	No
67	63	HL - Older	31,25	21,25	50	35	30	17	No
68	62	HL - Older	32,5	32,5	50	42,5	27	9	No
69	68	HL - Older	28,75	67,5	57,5	100	30	14	No
70	19	Young	6,25	5	9,5	12,5	-	13	No
71	19	Young	1,25	-2,5	5	7	-	13	No
72	20	Young	0	1,25	8,5	3,5	-	14	No
73	19	Young	12,5	10	17	13,5	-	13	No
74	69	HL - Older	21,25	23,75	38	55	29	12	No
75	56	HL - Older	36,25	23,75	58	45	30	11	No
76	19	Young	12,5	7,5	12,5	7	-	16	No
77	23	Young	0	-1,25	3,5	2,5	-	15	No
78	57	HL - Older	36,25	41,25	52,5	55	26	9	No
79	71	HL - Older	26,25	31,25	40	50	29	12	No

Supplementary Table 1. Anamnestic and HEARING CHARACTERISTICS OF PARTICIPANTS. Age, group, hearing thresholds for left and right ears (500Hz, 1kHz, 2kHz, 4kHz), perception threshold 50% (vocal audiometry) for left and right ears, MoCA scores, years of education, presence of Tinnitus.

**ITEM NUMBER**	LiCAS ITEM TRANSLATED AND ADAPTED IN ITALIAN	ENGLISH TRANSLATION OF LiCAS ITALIAN ITEM
**LiCAS - Self-Efficacy in Easy Conditions**		
**1**	Posso comprendere una conversazione con un'altra persona in un luogo tranquillo	I can understand a conversation with another person in a quiet place
**2**	Posso comprendere una conversazione con un'altra persona in un luogo tranquillo quando non riesco vedere il suo viso	I can understand a conversation with another person in a quiet place when I cannot see their face
**3**	Posso comprendere ciò che viene detto in una conversazione quando è pronunciato da una voce acuta, come quella di un bambino	I can understand what is said in a conversation when it is spoken in a high-pitched voice, like that of a child
**4**	Riesco a comprendere ciò che viene detto in TV la maggior parte delle volte	I can understand what is said on TV most of the time
**5**	Riesco ad ascoltare chi parla quando sono a una conferenza in una stanza grande e silenziosa	I can hear the speaker when I'm at a conference in a large, quiet room
**LiCAS - Self-Efficacy in Hard Conditions**		
6	Posso comprendere una conversazione con un'altra persona quando sta lavando i piatti e mi dà le spalle	I can understand a conversation with another person when they are washing the dishes and have their back to me
7	Posso comprendere una conversazione con qualcuno che parla sussurrando	I can understand a conversation with someone who speaks in a whisper
8	Posso comprendere una conversazione con un'altra persona quando c'è un rumore di fondo continuo, come quello di un ventilatore	I can understand a conversation with another person when there is continuous background noise, such as that of a fan
9	Posso comprendere conversazioni di gruppo quando c'è rumore di sottofondo	I can understand group conversations when there is background noise
**LiCAS – LoC - Hearing Loss Likelihood**		

1	Ogni tanto credo di avere un disturbo uditivo	I sometimes think I have an hearing loss
2	Non riesco ad ascoltare bene	I cannot hear well
3	Quando non riesco a capire qualcosa durante l'ascolto, sono portato a pensare di avere un disturbo uditivo	When I can not understand something while listening, I am apt to think I have an hearing loss
4	Indipendentemente da quanto lo voglio, non riesco a capire bene ciò che ascolto	I can not understand well what I am listening, even if I want to
5	Credo ci siano buone possibilità che io sviluppi un disturbo uditivo in futuro	I think there's a good chance I will develop an hearing loss
6	Se le persone intorno a me non mi aiutano, non riesco a comprendere bene ciò che viene detto in una conversazione	If the people around me do not help me, I can not properly understand what is being said in a conversation
**LiCAS - LoC - Control over Hearing Loss**		
7	Se mi impegno, posso migliorare le mie capacità di ascolto	If I work at it, I can improve my listening abilities
8	Se allenassi le mie capacità di ascolto con degli esercizi, riuscirei a capire meglio	If I would train my listening abilities through exercises, I would be able to understand better
9	Riesco a trovare delle soluzioni per migliorare le mie capacità di ascolto	I can find ways to improve my listening abilities

Supplementary Table 2 - LiCAS items

Negative/Maladaptive	Strategie maladattive
I get people to repeat by ignoring them	Quando non capisco quello che mi viene detto, porto la persona a ripetere ciò che ha detto ignorandola
I interrupt others when listening is difficult	Quando mi è difficile ascoltare, interrompo chi mi parla
I dominate conversations to avoid listening	Per evitare di ascoltare gli altri, cerco di dominare le conversazioni prendendo la parola
If someone irritated, I stop asking for repeat	Quando qualcuno a cui ho chiesto di ripetere qualcosa si irrita, smetto di chiedergli di ripetere
When I don't understand, I pretend I did	Quando non capisco, faccio finta di aver capito
When I don't understand someone, I ignore them	Quando non capisco qualcuno, lo ignoro
**Positive/Adaptive - Verbal**	**Strategie verbali**
If I don't understand repetition, I ask again	Se non capisco la prima ripetizione, chiedo alla persona di ripetere nuovamente ciò che ha detto
If I hear part, I only ask for repeat of rest	Se sento solo una parte di un messaggio, chiedo di ripetere solo ciò che non ho udito bene
I've asked family to get my attention first	Chiedo ai miei famigliari di attirare la mia attenzione prima di parlarmi
When I don't understand, I ask for repeat	Quando non capisco, chiedo di ripetere
I've asked friends/coworkers to get attention	Chiedo a colleghi/amici di attirare la mia attenzione prima di parlarmi
When someone speaks softly, I ask to speak up	Quando qualcuno parla troppo a bassa voce, chiedo di alzare la voce
**Positive/Adaptive - Non-verbal**	**Strategie non verbali**
Try to position myself to hear well	Cerco la posizione ideale per ascoltare meglio
When having trouble, pay close attention to face	Quando ho problemi a udire, mi focalizzo sul viso di chi mi sta parlando
If I can't hear, I'll move to another seat	Se non riesco ad udire, mi sposto in un altro posto
At parties, I try to stay in well lighted area	Alle feste, cerco di rimanere in un'area ben illuminata
When in group, try to sit where can hear better	Quando sono in gruppo, scelgo di sedermi in posti dove posso udire meglio
In background noise, position so less distracting	Quando c'è rumore di fondo, mi posiziono in modo che mi distragga il meno possibile
When having trouble, listen for main points	Quando ho problemi ad udire, mi concentro sulle informazioni principali del discorso
Try to watch person's face when speaking	Quando ho problemi ad udire, provo a guardare il viso della persona che parla

Supplementary Table 3 - COPING STRATEGIES FOR LISTENING CHALLENGES. All items from the three subscales are presented in the table. The left column contains the English version, and the right column provides the original Italian version used in the experiment.

Negative/Maladaptive	Strategie maladattive	Strategie maladattive (prospettiche)
I avoid social situations if I'll have problems	Evito le situazioni sociali a causa del mio problema di udito	Se dovessi avere un problema di udito in futuro, eviterò le situazioni sociali
I avoid conversing because of hearing loss	Evito di conversare a causa della perdita dell'udito	Se dovessi avere un problema di udito in futuro, eviterò di conversare
I avoid talking to strangers because of loss	Evito di parlare con gli sconosciuti a causa della perdita uditiva	Se dovessi avere un problema di udito in futuro, eviterò di parlare con gli sconosciuti
**Positive/Adaptive**	Strategie verbali	Strategie verbali (prospettiche)
When I have trouble, I remind of hearing problem	Quando ho problemi a udire, ricordo alle persone del mio problema di udito	Se dovessi avere un problema di udito in futuro, ricorderò alle persone del mio problema nelle situazioni in cui fatico a capire
When I don't understand, I explain hearing loss	Quando non capisco, spiego della mia perdita dell'udito	Se in futuro non dovessi capire a causa di una perdita uditiva, cercherò di spiegarlo alle persone con cui sto comunicando

Supplementary Table 3 - COPING STRATEGIES FOR HEARING LOSS. The table presents all items from the two subscales. The left column contains the English version, while the central and right columns show the original Italian versions used in the experiment. The central column lists strategies for participants who reported having a hearing impairment, and the right column lists prospective strategies presented to participants without reported hearing issues, addressing actions they might consider if they were to experience hearing loss in the future.

Group	word position				Mean
	First	Second	Third	Fourth	
Percent of correct words					
Young	0.42±0.12	0.40±0.06	0.82±0.10	0.69±0.09	0.59±0.07
Older adults - NH	0.51±0.13	0.46±0.09	0.80±0.10	0.72±0.14	0.62±0.08
Older adults - HI	0.50±0.12	0.48±0.14	0.73±0.10	0.67±0.14	0.60±0.08
Confidence					
Young	2.30±0.65	2.42±0.59	3.13±0.50	3.00±0.56	2.71±0.50
Older adults - NH	2.16±0.70	2.30±0.74	2.82±0.70	2.78±0.71	2.51±0.63
Older adults - HI	2.25±0.77	2.46±0.76	2.86±0.63	2.69±0.70	2.56±0.65
Area under the curve (metacognitive monitoring)					
Young	0.69±0.09	0.66±0.07	0.75±0.10	0.73±0.05	0.71±0.05
Older adults - NH	0.75±0.10	0.67±0.09	0.77±0.08	0.76±0.10	0.73±0.07
Older adults - HI	0.68±0.08	0.65±0.08	0.75±0.09	0.72±0.10	0.701±0.06

Supplementary Table 5 - HEARING IN NOISE TASK AND METACOGNITIVE MONITORING – YOUNG, OLDER ADULT WITH NORMAL HEARING and OLDER ADULTS WITH HEARING IMPAIRMENTS. Mean and standard deviation as a function of word position of percent of correct words, confidence and area under the curve. To the right, the mean values and the standard deviation of the three indices without splitting them as a function of word position.
